# Antioxidant Treatment and Induction of Autophagy Cooperate to Reduce Desmin Aggregation in a Cellular Model of Desminopathy

**DOI:** 10.1371/journal.pone.0137009

**Published:** 2015-09-02

**Authors:** Eva Cabet, Sabrina Batonnet-Pichon, Florence Delort, Blandine Gausserès, Patrick Vicart, Alain Lilienbaum

**Affiliations:** 1 Physiopathology of the striated muscle laboratory, Unit of Functional and Adaptive Biology, University Paris Diderot, Sorbonne Paris Cité, UMR CNRS 8251, Paris, France; 2 Biology and pathology of the endocrine pancreas laboratory, Unit of Functional and Adaptive Biology, University Paris Diderot, Sorbonne Paris Cité, UMR CNRS 8251, Paris, France; University of Vienna, Max F. Perutz Laboratories, AUSTRIA

## Abstract

Desminopathies, a subgroup of myofibrillar myopathies (MFMs), the progressive muscular diseases characterized by the accumulation of granulofilamentous desmin-positive aggregates, result from mutations in the *desmin* gene (*DES*), encoding a muscle-specific intermediate filament. Desminopathies often lead to severe disability and premature death from cardiac and/or respiratory failure; no specific treatment is currently available. To identify drug-targetable pathophysiological pathways, we performed pharmacological studies in C2C12 myoblastic cells expressing mutant *DES*. We found that inhibition of the Rac1 pathway (a G protein signaling pathway involved in diverse cellular processes), antioxidant treatment, and stimulation of macroautophagy reduced protein aggregation by up to 75% in this model. Further, a combination of two or three of these treatments was more effective than any of them alone. These results pave the way towards the development of the first treatments for desminopathies and are potentially applicable to other muscle or brain diseases associated with abnormal protein aggregation.

## Introduction

Protein aggregation represents a histopathological hallmark of a wide variety of human diseases principally affecting the central nervous system (Alzheimer's disease, Parkinson's disease, Huntington's disease, amyotrophic lateral sclerosis) and striated muscles (myofibrillar myopathies) (reviewed in [1]). Protein aggregates form when misfolded proteins interact abnormally—with each other or other proteins—and precipitate. This phenomenon results primarily from a disturbance in protein folding, but can occur after a failure in cellular protein quality control (PQC) mechanisms [2]. PQC is controlled first through molecular chaperones, including heat-shock proteins (HSPs). These chaperones bind to proteins with inappropriately exposed hydrophobic residues to inhibit their aggregation and allow refolding [3, 4]. However, chaperones may not repair aberrant proteins that will never properly fold or that are heavily post-translationally modified. In that case, chaperones send abnormal proteins to degradative pathways, typically the ubiquitin-proteasome (UPS) or autophagic systems [2], with the assistance of co-chaperone and ubiquitin-ligases. These complexes mark misfolded proteins for degradation with ubiquitin residues. The efficiency of the PQC system is influenced by many variables, including thermal, oxidative, viral, or age-related stress factors, which may either increase the production of abnormal protein and overload the PQC system, or target the PQC system itself and reduce its degradative and recycling capacity [5, 6]. If the PQC system is unable to cope with over-production of misfolded proteins, cell death ultimately results.

Myofibrillar myopathies (MFM, OMIM 601419), muscular diseases characterized by the presence of protein aggregates, have been defined at the histopathological level by a sarcoplasmic accumulation of granulofilamentous material, desmin-positive protein aggregates, and degenerative changes in the myofibrillar apparatus (reviewed in [7–10]). MFMs have been attributed to variations in genes encoding desmin, α-B crystallin, myotilin, ZASP (Z band alternatively spliced PDZ domain containing protein), filamin C, BAG3 (BCL-2-associated athanogene 3), plectin, FHL1 (four and a half LIM domains 1), and selenoprotein N. However, in 50% of patients the disease remains genetically uncharacterized [8, 11].

The *desmin* gene (*DES*) encodes the 53-kDa muscle-specific intermediate filament (IF) desmin. Desmin forms a sarcoplasmic network that encircles Z disks to connect them and binds them to the sarcoplasmic membrane and the nuclear lamina [12–14]. This three-dimensional organization is believed to enable alignment of the myofibrils and maintain the spatial relationship between the contractile apparatus and other structural elements of the muscle fibers [15, 16], thus minimizing shear stress during muscle contraction. *Desmin* mutations most often introduce single amino-acid substitutions in the central α-helical and highly conserved "rod" domain of the protein [7]. This domain is essential for polymerization of desmin into a correct and functional network, and therefore, aberrant desmin proteins can interfere with filament formation. In many cases, the desmin mutants cannot form functional networks [17, 18], but they are also capable of disrupting a preexisting filamentous network in a dominant-negative way [19]. In addition, perturbations of the cytoskeleton are associated with abnormal distribution of mitochondria and respiratory function abnormalities [20, 21].

One intriguing feature of MFMs resulting from mutations in *desmin* (also called desminopathies) is the adult onset of their progressive muscle phenotype, mainly between the second and fourth decade of life [7–10]. However, desmin is expressed early in the embryonic stage of human development [22], therefore desmin-related phenotypes would be expected earlier in life. One general hypothesis proposed to explain this discrepancy is the existence of compensating mechanisms involving the PQC system [23, 24] and muscle regeneration. When the PQC system (i.e., HSPs, UPS, and autophagy) becomes overwhelmed by sarcoplasmic aggregates and a general dysfunction of muscle fibers occurs, it leads to myofibrillar death. Then, muscle regeneration involving satellite cells, together with other muscular stem cells, is stimulated to renew muscle fibers. However, when this last compensating mechanism also fails (which can take decades), early exhaustion of the muscular precursors reservoir means muscular symptoms start to develop [25].

To date, no specific treatment exists for MFMs, and their progressive clinical course often leads to severe disability and premature death [7]. In accordance with the proposed model (mutation—PQC—muscle regeneration) to explain the long latency to symptom presentation in affected individuals, we hypothesized that stimulation of cellular mechanisms of defense, including the PQC system, may alleviate, if not abolish, the cellular burden caused by desmin aggregation. To test this, we used C2C12 cell lines expressing *DES* mutants to screen several pathways and pharmacological compounds that might stimulate cellular defenses, and found three ways to significantly lower the occurrence of desmin aggregation in these myoblasts. The findings suggest several novel therapeutic approaches for treating MFMs, which could have a critical impact on patient outcomes for a currently untreatable disease.

## Results

### Complex kinetics of mutant desmin aggregation

**Fig 1:**
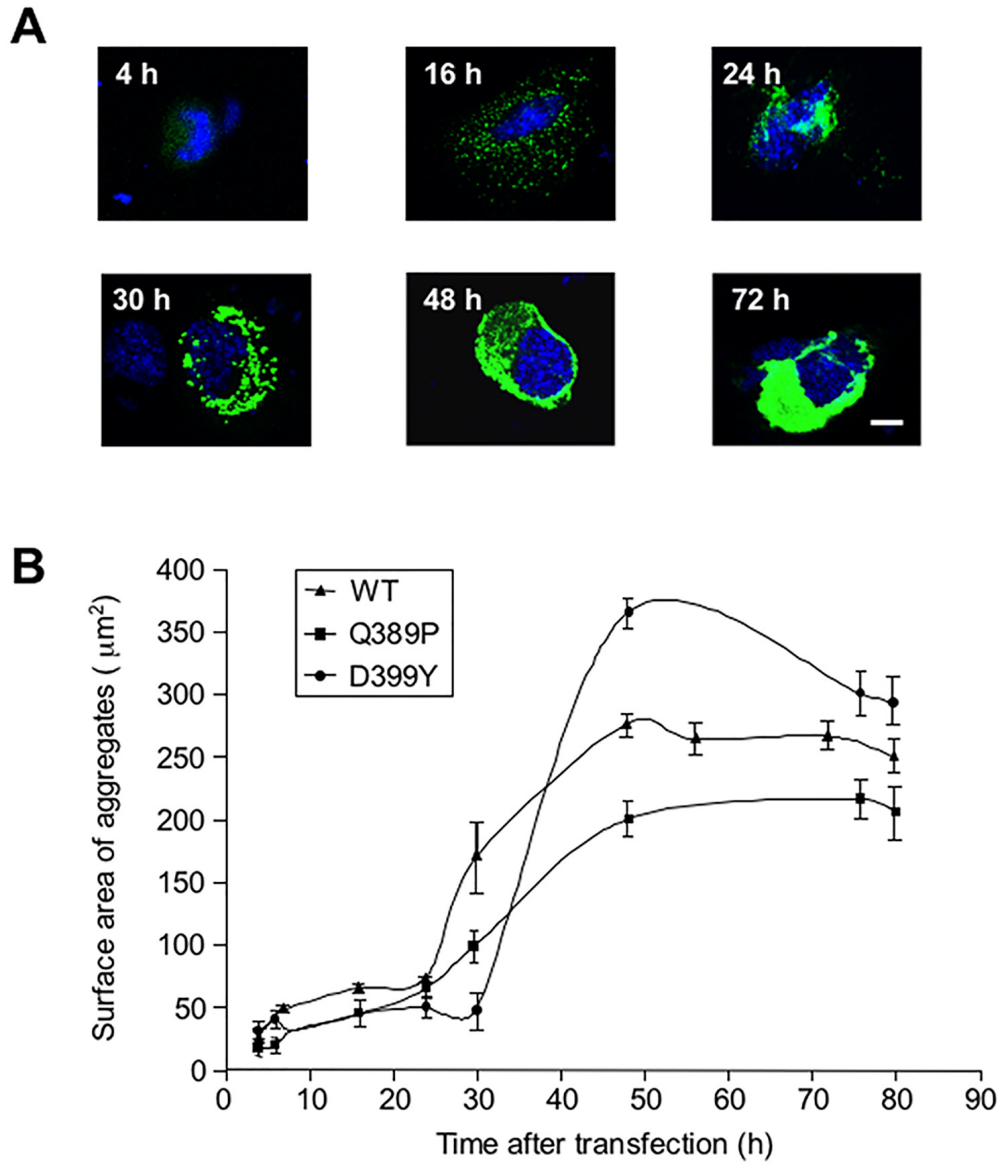
Kinetics of desmin mutant aggregation. (A) C2C12 murine myoblasts transiently transfected with an expression vector coding for a GFP-tagged desmin mutant D399Y were fixed at various times (4 to 80 h) following transfection. Photomicrographs are representative of the different phases of aggregation. Scale bar, 10 μm. (B) Surface areas of aggregates were measured on a panel of n = 15 cells for each time point and each experiment, in 5 independent experiments, and the mean value plotted against time, for desmin mutants Q389P, D399Y, and wild-type (WT). Error bars, s.e.m.

The appearance of desmin aggregates is characteristic of myofibrillar myopathies. To screen for pathways or pharmaceutical treatments affecting desmin aggregation, we chose to study a cellular model as the most convenient approach. Therefore, we used C2C12 myoblast cell lines that were transiently transfected with constructs expressing desmin mutants to generate aggregates. First, to better understand how desmin aggregates develop in muscle cells, we measured the growth kinetics of the aggregates. We chose to study desmin missense mutants p.Gln389Pro (Q389P) and p.Asp389Tyr (D399Y), which have strong aggregate production culminating at ~48 h after transfection with the mutant construct [19, 26]. We transiently transfected C2C12 myoblasts with GFP-tagged desmin mutant-expressing vectors, and measured the surface area of aggregates at various times between 4 and 80 h after the transfection. Representative pictures of aggregates taken at various times after the transfection are shown in Fig 1A. Puncta (GFP) were visible as soon as 4 h after transfection, accumulated at 16 h, and were compacted into one or two bigger aggregates situated near the nucleus at 24 h. When the larger aggregates developed, puncta were still being produced and accumulating (Fig 1A, 30 h). The large aggregate finally invaded the whole cytosol at 48 and 72 h. To quantify these results, surface areas of aggregates were plotted against time elapsed following transfection (Fig 1B). The curve displays a sigmoid shape for both mutants, as well as for the wild-type (WT) control, in accordance with other in vitro studies performed with different aggregative molecules, as well as with theoretical models [27]. There was a latency of 24–30 h, followed by a rapid 8-fold increase of aggregate surface area over about 20 hours, which later reached a plateau. Unexpectedly, the myc-Desmin WT construct showed a reduced latency phase and an early growing phase, but the myc-tagged desmin D399Y mutant confirmed the first results (S1 Fig). We concluded from this study that a period of latency with small aggregates precedes a phase of exponential-like growth in our model.

### Modulation of cell signaling pathways reduces desmin aggregation

We next reasoned that cells might mobilize protective mechanisms to avoid the production of aggregates detrimental to their correct functioning. We previously showed that production of desmin aggregates triggers the p38 mitogen-activated protein kinase (MAPK) signaling pathway [28]. Thus, we hypothesized that the first line of defense could be the modification of cell signaling pathways in response to, or closely related to, alterations occurring in the cytoskeleton. Here, we co-transfected GFP-tagged desmin mutant D399Y with kinase- or kinase-modulating protein-expressing constructs. Considering the results in Fig 1, we analyzed aggregates produced during the early latent period, 20 h after transfection. In addition, we investigated the late stationary phase, 48 h after transfection, to assess possible slow-acting mechanisms. Cells were fixed at the indicated times and the percentage of transfected cells with aggregates was recorded.

**Fig 2:**
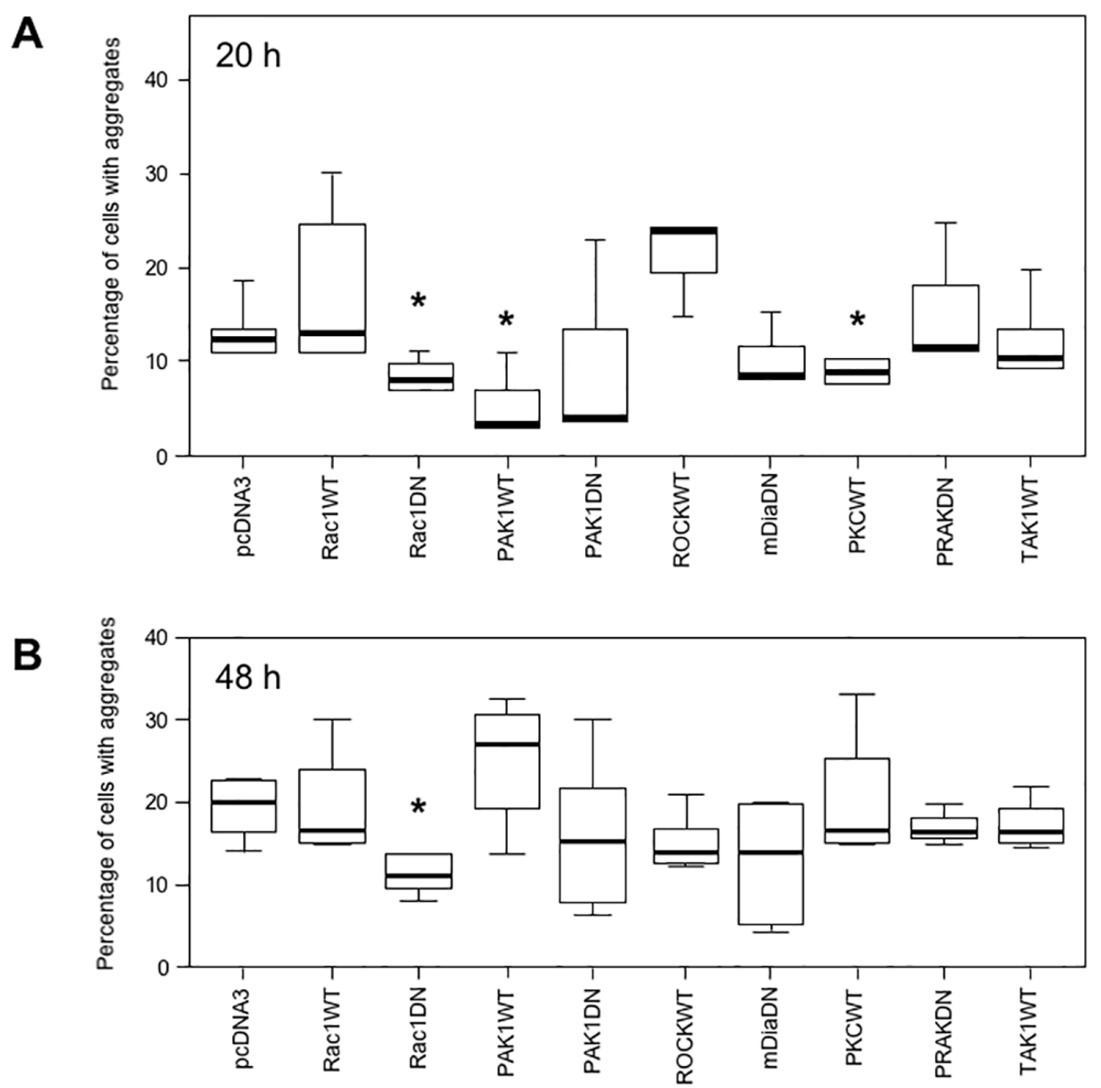
Modulation of cell signaling pathways related to the cytoskeleton reduces desmin aggregation. (A) C2C12 cells were co-transfected with a GFP-tagged desmin mutant D399Y and constructs coding for either wild type (WT) or dominant-negative mutant (DN) kinases or kinase-modulating proteins [i.e., Rac1, p21-activated protein kinase (PAK1), Rho kinase (ROCK), mammalian Diaphanous (mDia), protein kinase C (PKC), p38-regulated/activated protein kinase (PRAK) and transforming growth factor β activated kinase 1 (TAK1)]. At 20 h after transfection, cells were fixed and the total number of cells (n = 1500) and the number of cells with aggregates were counted. Experiments were performed 4 times. The percentage of cells with aggregates is displayed on a box plot graph (Tukey's diagram). Asterisk indicates a result statistically different from the control co-transfected with the desmin mutant and the empty vector pcDNA3 (p < 0.05 calculated with a non-parametric test). The black horizontal bar represents the median value; limits of the rectangle represent the first (25% lower values) and the third (75% lower values) quartiles, respectively. Error bars, Tukey's adjacent values. (B) Same treatment as for (A) except that cells were fixed 48 h after transfection.

Initial screens involved 31 constructs related to 18 kinases or kinase-modulating proteins, mainly involved in modulation of, or reponding to alteration occuring in, the cytoskeleton (S1 Table). These preliminary screens were reduced to 9 constructs related to 7 pathways, comprising the most efficient ones, which reduced by at least 30% the proportion of cells presenting aggregates. We checked that transfected cells correctly expressed most of the constructs used (S2 Fig). Several constructs, when co-transfected with desmin mutants, produced a significant reduction in the percentage of cells with aggregates at early stages: Rac1 dominant-negative (DN), PAK1 wildtype (WT) and PKC WT (20 h, Fig 2A). Results are presented for the D399Y desmin mutant, but similar data were obtained for the Q389P mutant (Fig 2 and data not shown). In addition, we verified that aggregate reduction was not resulting from cell death induced by the expression of construct-modulating cell signaling pathways (S3 Fig). The small GTPase Rac1 belongs to the Rac subfamily of RhoGTPases that activate protein kinases and regulate cell growth and cytoskeletal reorganization. Rac1 is specifically involved in cell cycle, cell-cell adhesion, and motility through reorganization of the actin network and epithelial differentiation [29]. Co-expression of Rac1 DN with the D399Y desmin mutant reduced the proportion of cells with aggregates by 35% (Fig 2A). In contrast, the Rac1 WT construct resulted in significant more cells with aggregates at 20 h. The effect of the Rac1 DN construct remained significant at 48 h after transfection (Fig 2B). PAK1 (p21-activated kinase) is a serine/threonine kinase that regulates cell morphology and motility and interacts with Rac1 [30]. The mean proportion of cells with aggregates was reduced by 74% in the presence of the PAK1 WT construct (Fig 2A). Protein kinase C alpha (PKC) is another serine/threonine kinase, belonging to a family of enzymes that respond to signals such as increases in the concentration of diacylglycerol (DAG) or calcium ions (Ca^2+^) [31]. The PKC construct reduced the percentage of cells with aggregates by 30% (Fig 2A). In addition, a control performed with the GFP-Desmin WT construct confirmed the effect of Rac1DN, PAK1 WT and PKC WT in the reduction of desmin aggregation (Fig A in S4 Fig), although other constructs were also able to reduce the percentage of cells harboring aggregates (ROCKWT and PRAKDN). To confirm these results obtained with GFP-tagged desmin constructs, we used in the same type of experiment a myc-tagged desmin WT as control and a myc-Desmin D399Y mutant (Fig B in S4 Fig). With both constructs, we found that Rac1DN, PAK1WT and PKCWT reduced significantly the proportion of transfected cells with aggregates. In fact, these three constructs were efficient in all configurations. We concluded from these results that modulating Rac1, PAK1, and PKC cell signaling pathways related to the cytoskeleton can reduce desmin aggregation.

### Tocopherols inhibit desmin aggregation

**Fig 3:**
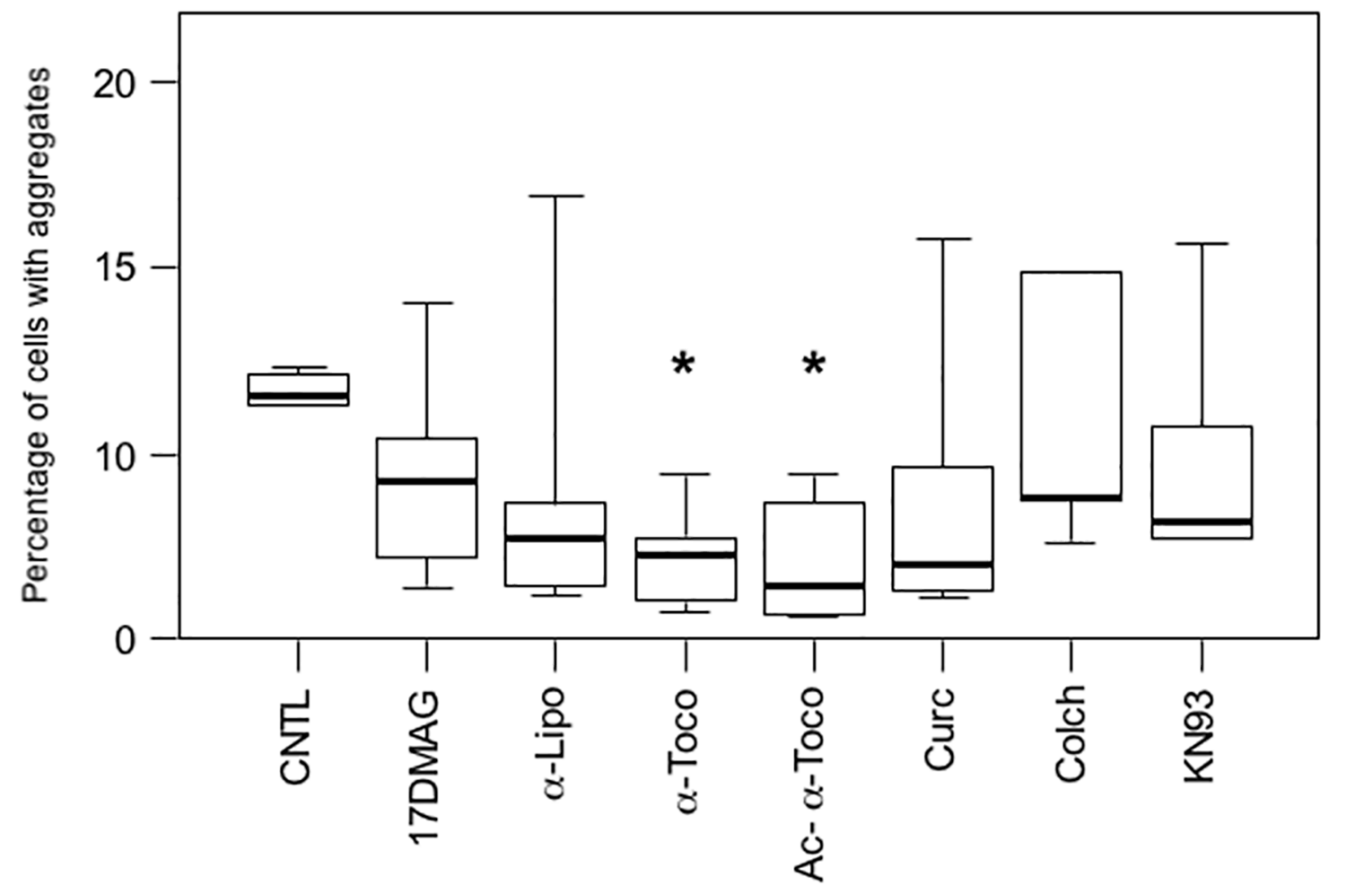
Treatment with vitamin E (α-tocopherols) inhibits desmin aggregation in muscle cells. Cells were transiently transfected for 4 h with the GFP-Desmin D399Y mutant and then treated with various pharmacological compounds for 16 h before fixation and counting for the percentage of cells with aggregates. 17-DMAG (20 μM) is a Geldanamycin derivative inducing heat-shock proteins; alpha-lipoic acid (α-Lipo, 300 μM), α-tocopherol (α-Toco, 300 μM), and acetyl-α-tocopherol (Ac-α-Toco, 100 μM) are antioxidants; curcumin (Curc, 5 μM) has anti-aggregative properties; colchicine (Colch, 10 μM) inhibits microtubule polymerization; and KN93 (10 μM) is an inhibitor of Calmodulin kinase II. The vehicle dimethylsulfoxide (DMSO) was used for the control (CNTL). The box plot represents the result of 4 independent experiments (n = 600). Significant differences from the control are indicated with asterisk (p<0.05 calculated with a non-parametric test).

We next investigated whether modulation of cell signaling pathways and cell protective mechanisms with pharmacological compounds could reduce desmin aggregation in our muscle cell model. We therefore screened more than fifty compounds involved in cell signaling pathways (S2 Table). Following this primary screening, we reduced the number of pharmacological compounds to the seven producing the strongest effect, reducing at least 30% of cellular aggregates, and tested them more extensively. These products were distributed into five main categories: heat shock protein (HSP) activator (17-DMAG, a derivative of Geldanamycin), antioxidant [alpha-lipoic acid (α-Lipo) and two forms of vitamin E, alpha-tocopherol (α-Toco) and *O*-Acetyl-alpha-tocopherol (Ac-α-Toco)], anti-aggregative [Curcumin (Curc)], inhibitor of cytoskeletal polymerization [Colchicine (Colch)], and modulator of calcium signaling (KN93). To test these, we transfected C2C12 myoblasts with the GFP-Desmin D399Y construct for 4 hours to limit the initial amount of mutant desmin produced. We expected that a moderate expression leading to a moderate production of aggregates could better reveal an effect of pharmacological products. The general tendency of all compounds tested for 16 h during the first round was to lower the proportion of cells with aggregates, but only one type of compound reached the statistical cut-off (Fig 3; marked by an asterisk on the graph (P < 0.05)). This product, alpha-tocopherol, belongs to the vitamin E family, which is known for its antioxidant properties. Alpha-tocopherol (α-Toco) reduced the proportion of affected cells by 65% and *O*-Acetyl-alpha-tocopherol (Ac-α-Toco), a more stable derivative, by 75%. These results were confirmed with GFP-Desmin WT and myc-tagged constructs (S4 Table). As reported for several other types of aggregative proteins, our results thus confirmed that antioxidant treatments reduce aggregation in various types of cells [32, 33].

To exclude a possible effect of the pharmacological compound, we used flow cytometry to determine whether alpha-tocopherols could be lethal for C2C12 cells and may even specifically kill cells expressing desmin mutants and bearing aggregates. The analysis was performed on cells transiently transfected with the desmin mutant GFP-Desmin D399Y (S5 Fig). We did not find any specific toxicity associated with the expression of desmin mutants and production of aggregates in cells treated with alpha-tocopherols.

### Stimulation of autophagy inhibits desmin aggregation

We then decided to check whether induction of autophagy, a proteostasis mechanism able to remove aggregates, could reduce desmin aggregation in muscle cells. To stimulate autophagy in C2C12 myoblasts, we tested several products described in the literature to trigger the process [34] (S3 Table). For this purpose, we monitored the level of microtubule-associated proteins LC3-I and LC3-II, which are the main indicators of autophagy as essentials components of the autophagic vesicles called autophagosomes [35]. LC3-I matures and is processed into the LC3-II form by cleavage and addition of phosphatidylethanolamine. The quantity of LC3-II formed is generally considered as proportional to the intensity of autophagy [36]. We found that PP242 was the most efficient inducer of autophagy in myoblasts, producing a 2- to 3-fold activation over the control samples (S3 Table and S6 Fig).

**Fig 4:**
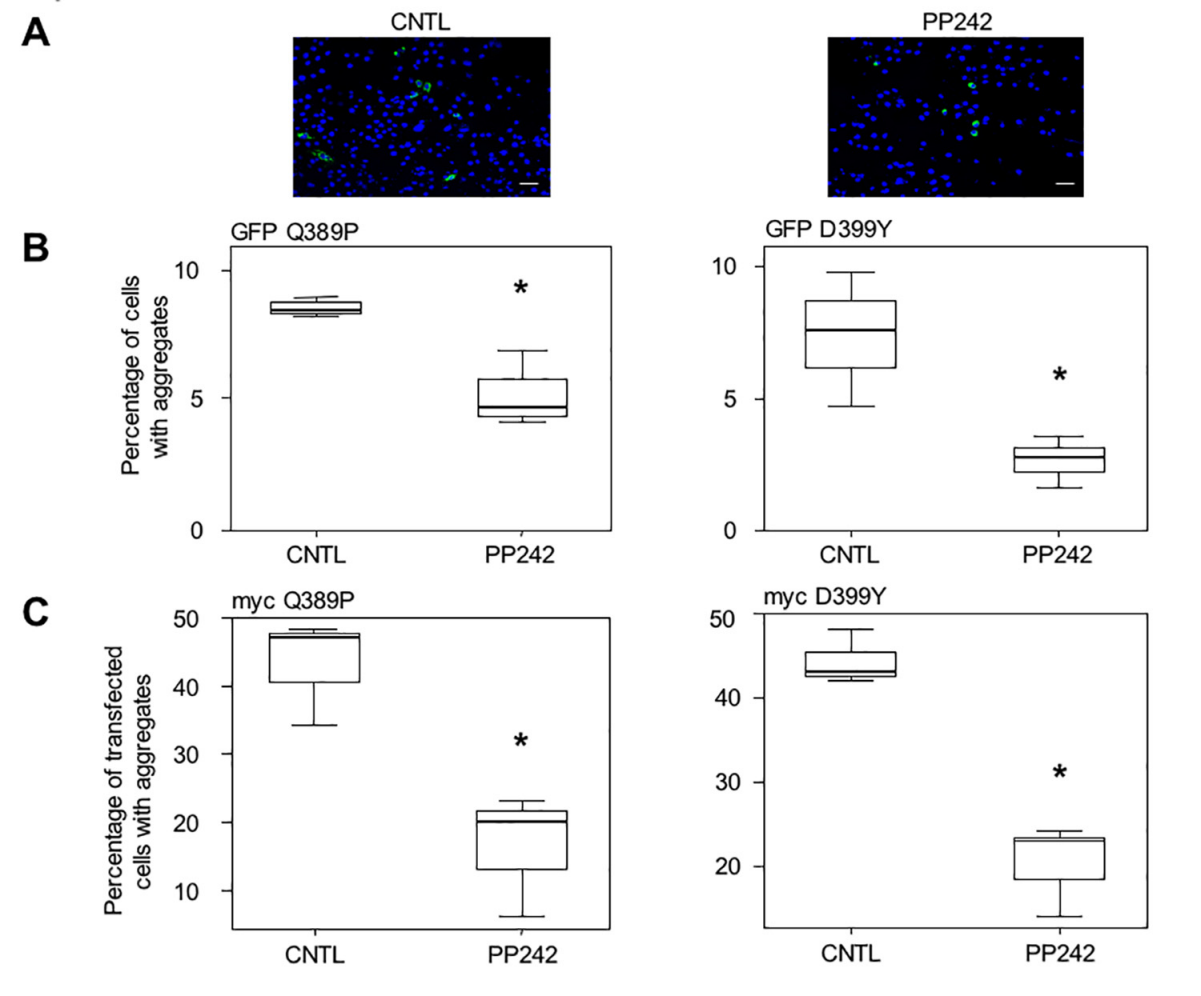
Stimulation of autophagy with PP242 reduces desmin aggregation in myoblasts. (A) C2C12 cells were transiently transfected for 4 h with GFP-Desmin D399Y mutant, washed, and treated for 16 h with PP242 (10 μM). They were fixed and several fields photographed. A typical field is displayed. Green dots are cell aggregates and blue dots are cell nuclei visualized with DAPI. Cells from the control panel (CNTL) were treated with DMSO. Scale bar, 30 μm. (B) Quantification of 3 independent experiments (n = 1200 total cells for each condition in each experiment). The left panel displays results obtained with the GFP-Desmin Q389P construct, and in the right panel, from GFP-Desmin D399Y transfections. (C) Same as for (B), except that myc-Desmin Q389P (left panel) and myc-Desmin D399Y (right panel) constructs were used. Cells were transiently transfected for 4 h with these constructs, washed, and treated 16 h with PP242 (10 μM) or DMSO as solvent (CNTL). They were fixed and then processed for immunocytochemistry with an anti-myc antibody. Numbers of cells with aggregates per field and number of myc-positive cells without aggregates but displaying a normal desmin network were counted. The percentage of cells with aggregates among all myc-positive cells was calculated, taking into account 5 fields per experimental condition (n = 1400) in 3 independent experiments. Transfection efficiency was 25%. Significant differences from the control are indicated with asterisk (p<0.05 calculated with a non-parametric test).

As expected, treatment with PP242 (10 μM) for 16 h following transfection reduced the number of cells harboring aggregates, as can be seen in photomicrographs in Fig 4A. Quantification of the percentage of cells with aggregates among the total cell population demonstrated that the proportion of cells with aggregation was reduced by 44% for GFP-Desmin Q389P and by 63% for GFP-Desmin D399Y (Fig 4B). Similar results were obtained with the GFP-Desmin WT control (S4 Table). We further confirmed these results by using other desmin constructs tagged with a myc epitope and containing the same mutations as described above. With these constructs, we could not only visualize cells containing aggregates, but also cells positive for myc with a desmin network that was identical to the endogenous network produced in normal cells, but without aggregates (data not shown). Both mutants Q389P and D399Y presented this network, suggesting that mutant desmin molecules can be incorporated at a low level in the desmin network without necessarily creating aggregates. Notably, cells with a normal network and no aggregates were less frequently observed using the GFP tag, likely due to steric hindrance. We added PP242 to cell culture, in the same manner as described for Fig 4B, and revealed the expression of myc-tagged desmin mutants. Counting stained cells with aggregates and positive cells with normal network and no aggregates, we confirmed the previous result for PP242: this inducer of autophagy reduced the proportion of transfected cells with aggregates by 57% (myc-Q389P) and 47% (myc-D399Y) among myc-positive transfected cells (Fig 4C). Similar results were obtained with the myc-Desmin WT control (S4 Table).

We therefore concluded that PP242 is efficient in reducing the number of desmin aggregates in myoblast cells. Altogether, these results suggest that activation of autophagy markedly reduces the number of aggregates produced by expression of desmin mutants by around 50% in myoblasts.

Next, we checked whether PP242 could have a toxic effect on cells, and more precisely on transfected cells containing aggregates. Cell death was assessed in cells transiently transfected with the GFP-D399Y construct after treatment with PP242 using a cell fluorimeter (S7 Fig). A moderate global toxicity was associated with treatment of C2C12 cells with PP242 (from 5% of the total cell population treated with DMSO to 13% of those treated with PP242). However, the proportion of cell death among transfected cells (GFP+) was not higher with PP242 (1.2-fold) compared to treatment with the solvent alone (1.4-fold). Therefore, cell death of GFP-Desmin D399Y–expressing cells does not explain the specific reduction of aggregation in these cells.

### Stimulation of autophagy inhibits stress-induced desmin aggregation

**Fig 5:**
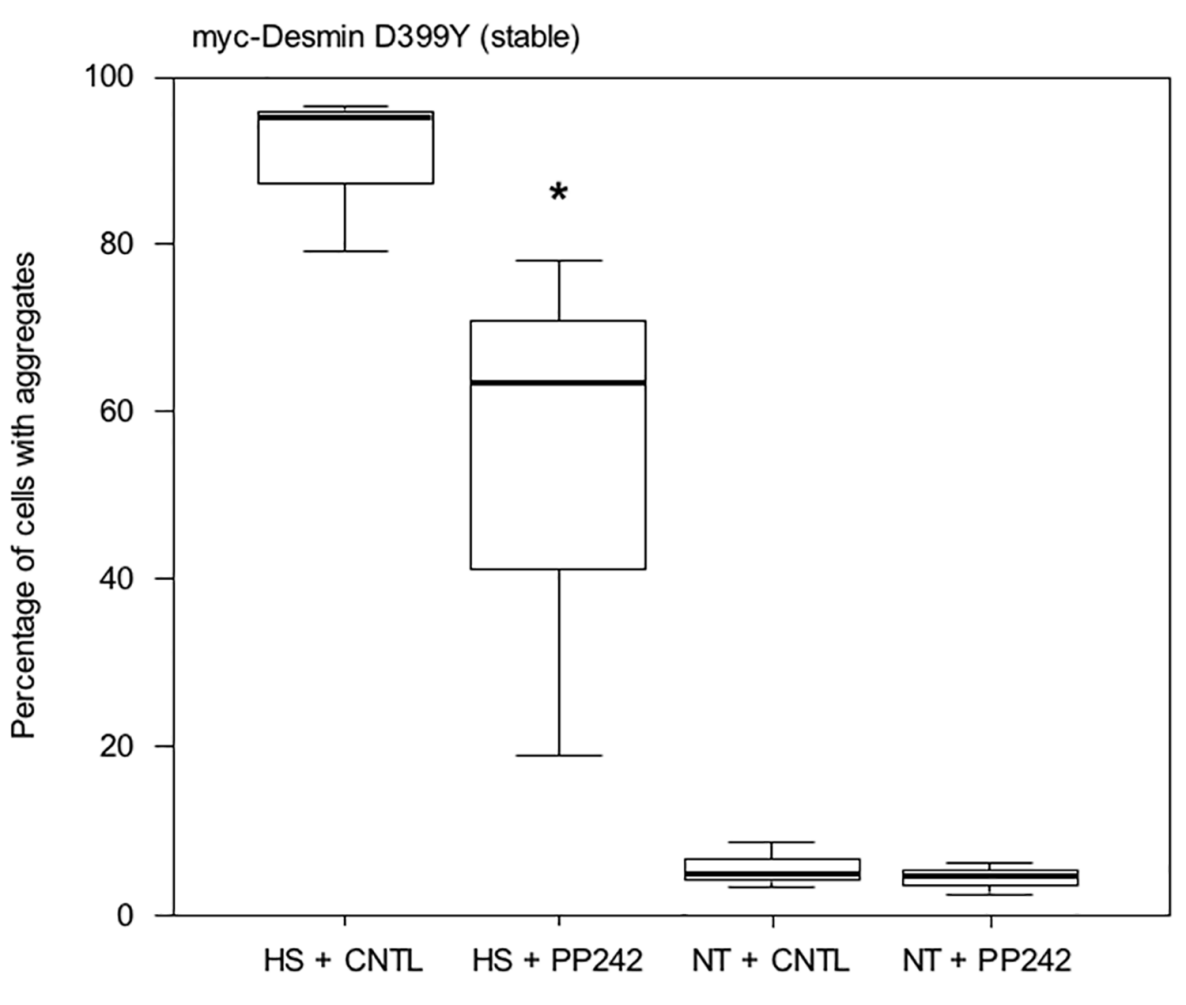
PP242 reduces aggregation in stressed C2C12 cells stably expressing the myc-Desmin mutant D399Y. C2C12 cells stably transfected and expressing the myc-Desmin D399Y mutant construct under a doxycycline-inducible promoter (Tet-ON system), 48 h after induction by doxycycline, were treated with PP242 or DMSO for 16 h. Cells were then subjected to heat-shock at 42°C for 2 h (HS), the medium was changed, and cells were incubated in normal growth medium for 24 h. Cells were then fixed and aggregates were counted under a microscope (9 fields, n = 1200 total cells in 3 independent experiments). As a control, cells not heat-shocked (NT) were also analyzed. Asterisk indicates a result statistically different from the control (p < 0.05 calculated with a non-parametric test).

We then confirmed the previous result in a stable cell line. We have previously described Doxycycline-inducible cell lines (DesD399Y clone C26) in which the expression of a myc-desmin D399Y mutant construct can be triggered [33]. This stable cell line corresponds to a more physiological model, but they produce only very few aggregates under standard conditions. Therefore, a stress (i.e., thermic, osmotic, or oxidative) must be applied to trigger desmin aggregation. We applied thermic stress for 2 h at 42°C following 16 h of PP242 treatment in the presence of Doxycycline and placed then cells in normal culture medium for 24 h. Results are shown in Fig 5. Interestingly, almost all cells (95%) exhibited aggregates after heat-shock (HS + CNTL). Treatment with PP242 lowered the number of cells with aggregates by 33% in heat-shocked cells (HS + PP242). Only around 5% of unstressed cells displayed aggregates, and this percentage was almost unaffected by the addition of PP242.

### Modulation of cell signaling pathways stimulates autophagy

**Fig 6:**
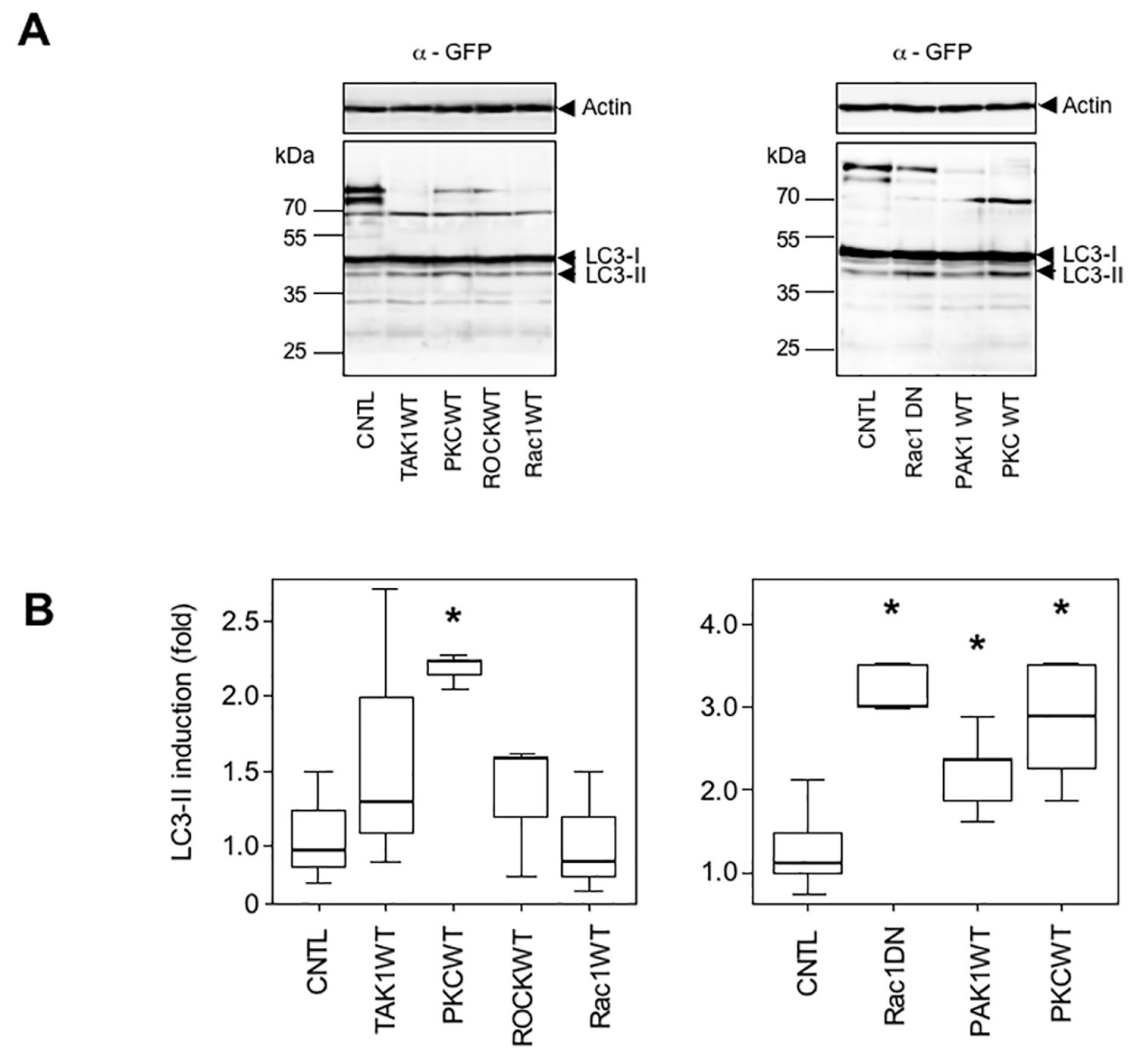
PKC WT, PAK1 WT, and Rac1 DN induce autophagy in C2C12 myoblasts. (A) LC3 levels in C2C12 cells expressing several constructs. Cells were transfected for 16 h with TAK1 WT, PKC WT, RhoK WT (ROCK), Rac1 DN, PAK1 WT or pcDNA3 (CNTL), together with a GFP-LC3 construct to measure autophagy in co-transfected cells. Cells were then lysed and GFP-LC3-II levels quantified in Western blots to estimate autophagy intensity. A representative result is shown in panel A. Actin was used as loading control. Levels of activation of autophagy were obtained by quantification of the LC3-II (anti-GFP antibody) band normalized to actin (anti-actin antibody). The control value (CNTL) was assigned to 1.0. (B) Statistical analysis of activation of autophagy. Experiments shown in (A) were repeated 3 times and displayed as dot plots. Asterisk indicates a result statistically different from the control co-transfected with the empty vector pcDNA3 (p < 0.05 calculated with a non-parametric test).

Having shown that stimulation of autophagy efficiently reduces aggregate production in muscle cells, we asked if PAK1 WT, PKC WT, or Rac1 DN constructs that reduced the number of aggregates (Fig 2) could ultimately activate the autophagic pathway. We co-transfected a GFP-LC3 construct with PAK1 WT, PKC WT, or Rac1 DN expression vector (Fig 6). The GFP-LC3 construct enables monitoring of autophagy more precisely in co-transfected cells, which have a high probability (generally estimated around 0.8) of co-expressing the PAK1 WT, PKC WT, or Rac1 DN construct. We first verified that the GFP-LC3 construct was properly working in our cells (S8 Fig). Following co-transfection, we found that, surprisingly, PAK1 WT and PKC WT were able to induce more than 2-fold the level of LC3-II, reflecting the number of autophagic vesicles [62]. With regard to Rac1 DN, the induction of autophagy was less surprising as a relationship with mTOR and autophagy has already been reported [37]. In contrast, other constructs that did not play a role in aggregate reduction (TAK1 WT, ROCK WT, and Rac1 WT) did not significantly modify the level of autophagy.

In conclusion, we show here that all three constructs modulating cell signaling pathways related to the cytoskeleton, and reducing the rate of aggregation, are also inducing autophagy. However, it cannot be excluded that other mechanisms are involved to reduce aggregation levels (see discussion below).

**Fig 7:**
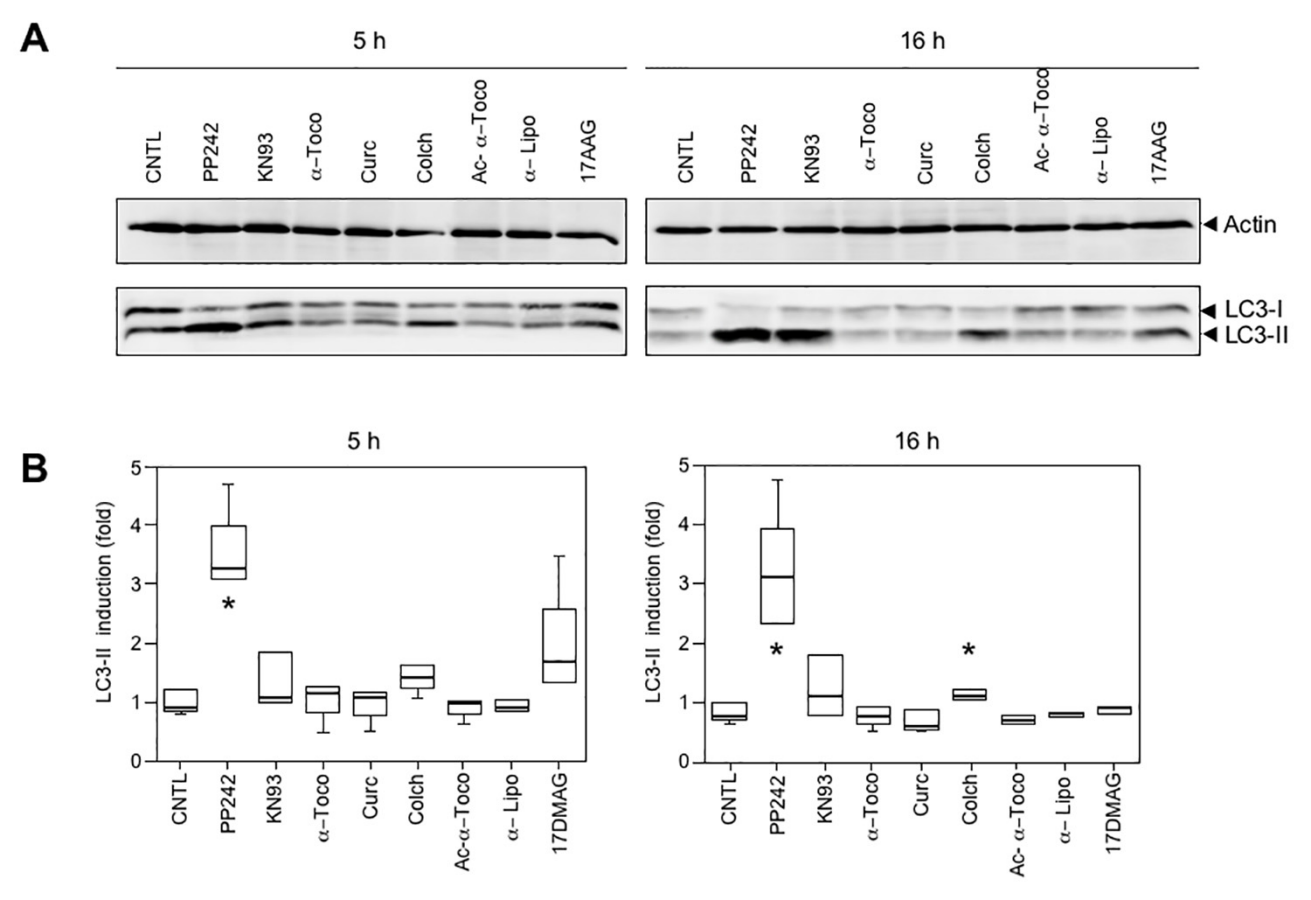
No induction of autophagy by pharmaceutical compounds. (A) Representative Western blot stained with anti-actin (upper panel) and anti-LC3 (lower panel). C2C12 cells were treated with PP242 (10 µM), KN93 (10 μM), α-tocopherol (α-Toco, 300 μM), curcumin (Curc, 5 μM), colchicine (Colch, 10 μM), *O-acetyl*-α-tocopherol (Ac-α-Toco, 100 μM) and 17-DMAG (20 μM) for 5 h or 16 h. Cells were then lysed and analyzed by Western blotting for actin and LC3-II levels (arrowheads). The ratio between intensity of LC3-II band and actin band was calculated, and the ratio for the control lane (CNTL) set to 1.0. (B) Box plot representation of 3 independent experiments and statistical analysis. At 5 h (left panel) only PP242 showed a significant induction of autophagy (p < 0.05 with a non-parametric test), indicated by an asterisk.

We also wished to determine if pharmacological products studied in Fig 2 could stimulate autophagy. We therefore treated cells with various compounds for 5 h or overnight (16 h) to ensure for efficient stimulation and monitor LC3 maturation. Results shown in Fig 7 revealed that no induction could be found beyond that of the positive control PP242. Fig 7A shows one representative result taken from three Western blots; Fig 7B is a compilation of three independent experiments. Even with longer treatment (16 h, Fig 7B right panel), the only induction noted was by colchicine. In fact, colchicine is an inhibitor of microtubule polymerization, which reduces transport of autophagosomes and lysosomes, therefore blocking the autophagic process at its endpoint [38].

### Cooperation between autophagy and tocopherols further inhibits desmin aggregation

**Fig 8:**
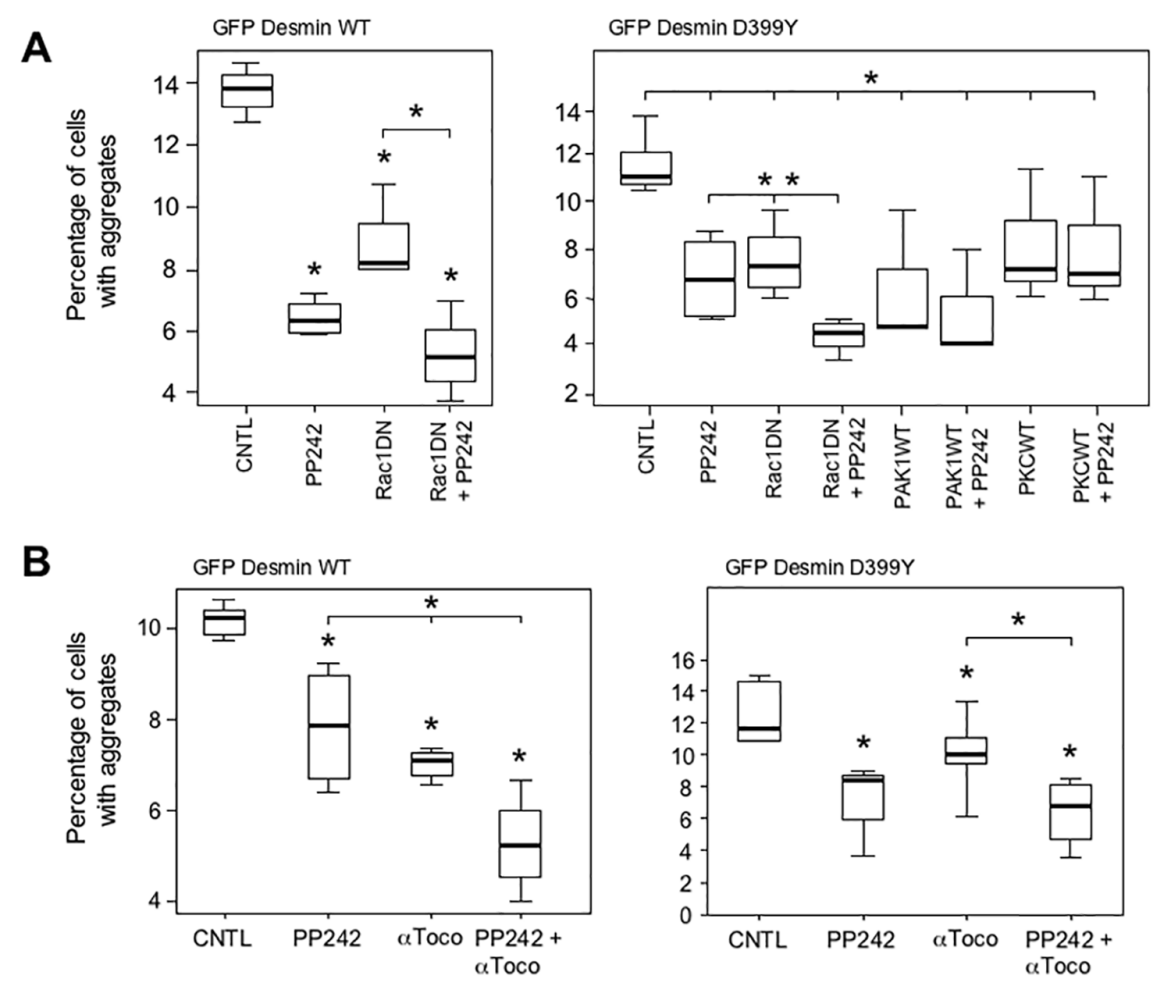
Autophagy inducers and antioxidants cooperate to reduce aggregation in C2C12 myoblasts. (A) Expression of Rac1 DN protein and treatment with PP242 cooperate to reduce desmin aggregates. C2C12 cells were co-transfected with the GFP-Desmin WT- (left panel) and D399Y- (right panel) expressing vectors and Rac1 DN, PAK1 WT, PKC WT, or pcDNA3 (CNTL and PP242) for 4 h. Cells were washed and subsequently incubated with PP242 (5 μM) or DMSO for 16 h. Cells were then fixed and the numbers of cells with aggregates were counted under a microscope (n = 100). A box plot representing 3 independent experiments is shown. Statistical analysis showed significant differences from pcDNA3 (p < 0.05 with a non-parametric test) as indicated by an asterisk. A significant difference for Rac1 DN + PP242 treatment versus either PP242 or Rac1 DN alone is indicated by an asterisk (p < 0.01) over an horizontal bar. (B) PP242 and α-tocopherols cooperate to reduce desmin aggregation. C2C12 cells were transiently transfected with a GFP-Desmin WT (left panel) or D399Y (right panel) vectors for 4 h. They were washed and then treated with PP242 (5 μM), α-tocopherol (α-Toco, 150 μM), or both for 16 h. The box plot represents 3 independent experiments. An asterisk indicates a significant difference from control at p < 0.05, and an asterisk above an horizontal bar indicates a significant difference between the double and simple treatments (p < 0.05).

Having shown that different treatments reduce desmin aggregation in muscle cells, we next asked whether applying two different treatments could further reduce the percentage of cells with aggregates. To test this, we used transient co-transfection of GFP-desmin constructs with Rac1 DN, PAK1 WT, or PKC WT expressing vectors for 16 h, combined with treatment with alpha-tocopherols or PP242. In a second type of test, using only pharmacological products, cells were transfected for 4 h with GFP-Desmin WT, Q389P or D399Y mutants and then treated with alpha-tocopherol and PP242 for 16 h. The most promising combinations are shown in Fig 8. Surprisingly, the most significant effect was induced by the combination of Rac1 DN expression and PP242 treatment, which cooperated to reduce the percentage of cells with aggregates by more than the effect of either individually (Fig 8A). PP242 reduced the percentage of cells with D399Y aggregates by 39%, Rac1 DN by 34%, and the combination by 60%. We also found that co-treatment with PP242 and alpha-tocopherol (α-Toco) produced a cooperative effect for both WT (left panel) and D399Y mutant (right panel)(Fig 8B). In particular, the individual treatments reduced the percentage of cells with D399Y aggregates by between 14 and 28%, while the combination of PP242 and α-Toco reduced the percentage of cells with aggregates by 45%. In that case, an antioxidant molecule combined with a pro-autophagic treatment cooperated to reduce the proportion of cells with aggregate. The effect, though significant, was less marked compared to that obtained with Rac1 DN and PP242 (60%). These results were confirmed using myc-tagged constructs in similar experiments (S9 Fig).

**Fig 9:**
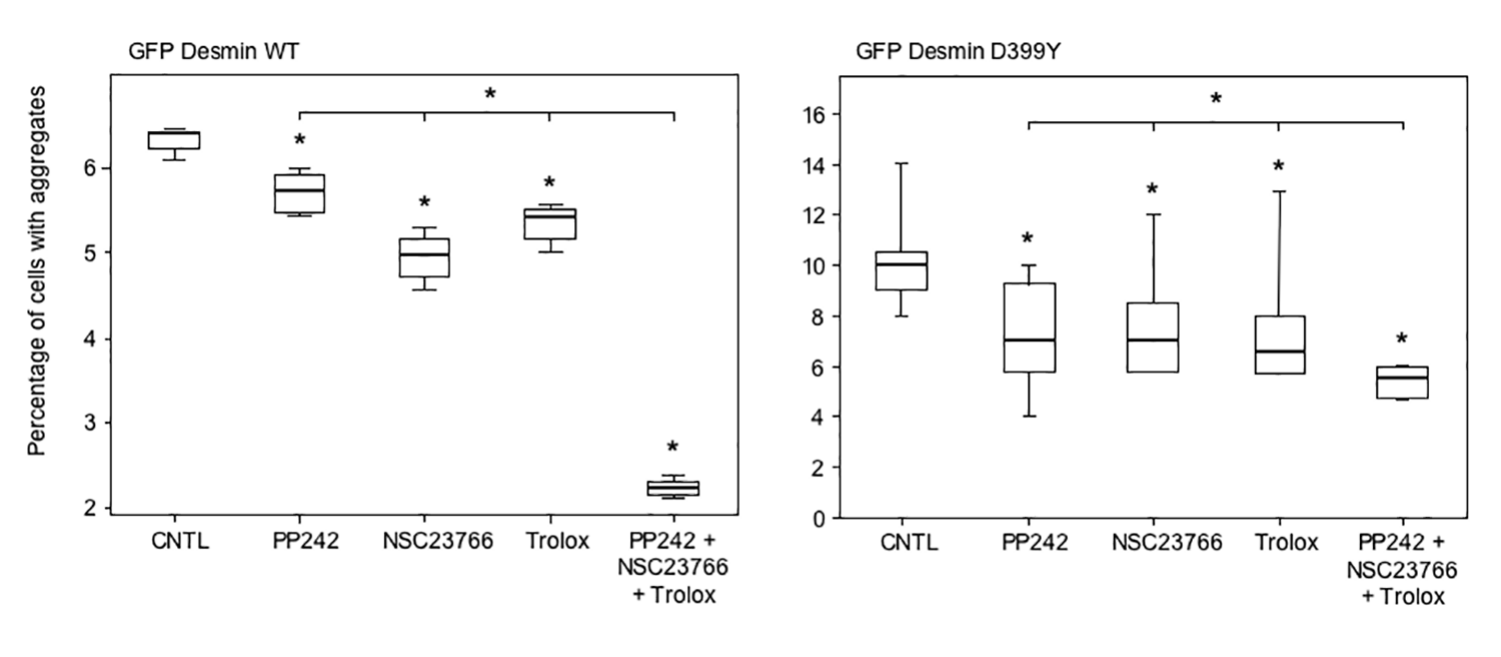
Triple treatment with therapeutic drugs cooperates to reduce desmin aggregation. C2C12 cells were transiently transfected with the GFP-Desmin WT- (left panel) or D399Y- (right panel) expressing vectors for 4 h. Cells were washed and subsequently incubated with PP242 (5 μM), NSC23766 (50 µM), Trolox (200 μM), all three compounds, or DMSO (CNTL) for 16 h. Cells were then fixed and counted under a microscope for the percentage having aggregates. A box plot representing 3 independent experiments is shown (n = 100). Statistical analysis with a non-parametric test revealed significant differences indicated by a simple asterisk (p < 0.05), and an asterisk above an horizontal bar indicates a significant difference between the double and simple treatments (p < 0.05).

To further investigate whether these cooperative effects between different anti-aggregative treatments described in Fig 8 could lead to a therapeutic treatment, we used an inhibitor of Rac1, NSC23766, that has been described as inducing autophagy and is also used as an anti-cancer drug. In addition, α-tocopherols are reportedly replaceable by a more soluble compound called Trolox [39]. Consequently, we transfected C2C12 myoblasts with the GFP-Desmin WT or D399Y constructs for 4 h, and treated cells with PP242, NSC23766, Trolox or all three products together for 16 h (Fig 9). PP242, NSC23766, and Trolox administered individually reduced desmin aggregation by 30%, 30%, and 35%, respectively with the D399Y mutant (right panel). As expected, the simultaneous treatment with all three compounds significantly reduced aggregation by 45%, or 10 to 15% more than any individual treatment (right panel). The cooperative effect was even more marked with the myc-tagged WT desmin construct (left panel), with a further reduction of ~ 50% in desmin aggregation compared to each single treatment. We checked that this treatment did not induce massive cell death that could explain the reduction of desmin aggregation (S10 Fig). The triple treatment produced 11.7% total cell death. Analyzing specifically transfected cells (GFP+), we found that the cell death level was similar, with 10.4% for the simultaneous use of PP242, NSC23766, and Trolox. We confirmed with myc-tagged WT and D399Y desmin constructs the results shown above (Fig A in S11 Fig). In addition, we used the inducible cell line described in Fig 5 and expressing the myc-Desmin D399Y mutant to confirm these results. Although stable clones were sensitive to the triple treatment, when cumulated with the heat-shock required to trigger desmin aggregation, the simultaneous use of PP242 and Trolox was 10% more efficient than each product used alone (Fig B in S11 Fig). Together, these results demonstrate that clinically useful pharmaceutical products can be combined to reduce desmin aggregation, which could reduce the cellular burden in myofibrillar myopathies.

In conclusion, we show in this study that manipulating cell signaling pathways (i.e., PAK1, Rac1, PKC, or NSC23766), activating autophagy (mTor inhibitor/PP242), and using antioxidants (α-tocopherols or Trolox) efficiently reduces up to 75% of aggregation of desmin intermediate filament mutants in muscle cells (summarized in S4 Table). In addition, combining different treatments further reduces aggregation, offer a promising avenue for therapeutic treatments of desminopathies.

## Discussion

Myofibrillar myopathies represent a group of neuromuscular diseases, generally characterized by a severe myopathy, and associated with cardiomyopathy in 15 to 30% of cases. Weakness and atrophy of the distal muscles of the lower limbs, which then progress to the hands and arms and finally reach the trunk and the face, are the common symptoms of this heterogenous group of muscular dystrophies [7–10]. However, in some patients, cardiomyopathy may precede muscle weakness [23, 40]. Desmin-positive protein aggregates as well as granulofilaments and electron-dense amorphous materials are the morphological hallmarks of desmin myo- and cardio-pathies. That lack of any specific treatment for MFMs has prevented improvements in patient outcomes [41].

Seeking to inhibit desmin aggregation by stimulating cellular defenses against protein aggregation to identify possible therapies for desminopathies, we screened several approaches to activate anti-aggregative cellular mechanisms. By transfecting myoblast cells for only 4 h with constructs expressing desmin mutants, we sought to obtain a moderate overexpression before applying various treatments. First, we found that a dominant-negative form of Rac1 and wild-type forms of PAK1 and PKC reduced the proportion of muscle cells with aggregates. Second, we screened several pharmacological compounds acting on a large variety of cellular mechanisms, and selected alpha-tocopherols, belonging to the vitamin E family, as the product most efficiently inhibiting desmin aggregation. Third, we targeted autophagy, a process that could ultimately remove aggregates, and selected a powerful inducer, PP242, that was able to reduce by more than 50% the burden of cellular desmin aggregates. In addition, a combination of two of these treatments was more effective than each alone.

The effect of the Rac1 DN protein appears straightforward, because Rac1, a member of the Rac subfamily of the Rho family of small signaling GTPases, is involved in the activation of mTor kinase complexes 1 and 2 (mTorC1 and mTorC2) [37]. Co-expression of a dominant-negative form of Rac1 therefore lowers mTor activity, thus stimulating autophagy. Indeed, it has been reported that inhibition of Rac1-mTor signaling pathway in coronary arterial myocytes enhances autophagosome formation [42]. It is surprising, however, that Rac1 DN cooperates with PP242, a strong inducer of autophagy in our cells. These two effectors may act either on different levels of the autophagic pathway, or on another unknown mechanism to reduce desmin aggregation. For example, other kinases, in particular PKC and PAK1, are able to phosphorylate desmin, which reduces desmin polymerization in vitro [43], and which may also reduce its aggregation. Phosphorylation along with other post-translational modifications, such as ADP-ribosylation and glycation, induce disassembly of desmin, at least in vitro [43]. However, a preliminary study on desmin by isoelectrofocusing did not reveal such a general phosphorylation (data not shown). Further work should clarify this point.

PAK1 is a crucial regulator of a variety of cellular processes, such as motility, cell division, gene transcription, and apoptosis [44]. It is still poorly understood how, at a molecular level, PAK1 operates in such diverse functions. PAK1 is a downstream effector of Rac1 and CDC42 [45, 46]. It is therefore not clear why inhibition of Rac1, but stimulation of the PAK1 pathway, induces autophagy. Interestingly, it has been shown that PAK1 is activated by the mTor–p70S6 kinase pathway [47]. One hypothesis explaining this dissociation between Rac1 and PAK1 is that PAK1 may be involved in a negative feedback loop. An excess of PAK1 could downregulate mTorC1 activity, and thus stimulate autophagy. In addition, PAK1 has been reported to have kinase-independent functions that have been ascribed to its scaffold capacity [44]. This fact may also explain how an excess of PAK1 could facilitate the assembly of multiple protein signaling molecules and control autophagy.

The protein kinase C (PKC) family of serine/threonine kinase regulates diverse cellular functions, including cell death, proliferation, and survival. In particular, PKC∂ mediates the activation of autophagy and apoptosis in response to stress conditions such as hypoxia, oxidative stress, nutrient starvation, or endoplasmic reticulum (ER) stress. PKC∂, which is the PKC isoform predominantly expressed in skeletal muscle, is an ER stress sensor required for autophagy activation in C2C12 myoblasts [48]. Interestingly, cardioprotection against ischemia-reperfusion injury also requires PKC∂-dependent induction of autophagy [49]. The mechanism by which PKC∂ stimulates autophagy is not clear. It has been previously described that PKC∂ activates JNK, which in turn phosphorylates BCL-2 bound to Beclin1, a BH3 (Bcl-2 homology) domain–containing protein demonstrated as essential for autophagy. Upon phosphorylation, BCL-2 dissociates from Beclin1, which allows this protein to participate to autophagy [50]. We used a PKCα expression vector in our experiments. Overexpression of PKCα may eventually saturate and stimulate several PKC pathways, including PKC∂ ones. In support of the role of PKCα in autophagy, it has been demonstrated that the classical isoform PKCα is also able to stimulate autophagy in response to oxidative stress both in mouse embryonic fibroblasts and in primary hepatocytes. Further work should help to decipher how these cell signaling mechanisms play a role in autophagy regulation.

We demonstrated here that α-tocopherol and acetyl-α-tocopherol, a more stable molecule, reduce the proportion of cells with desmin aggregation by 65 to 75%, and similar findings were produced in a stable cell line. In a previous work, we showed that another powerful antioxidant compound, N-acetyl-L-cysteine, prevents stress-induced desmin aggregation in the DesD399Y clone C26 stable cell line [33]. Muscle cells are particularly prone to accumulating oxidative damage to DNA, lipids, and proteins over time ([51] and references therein). Among several post-translational modifications, desmin was demonstrated to be a major target of oxidation and nitration in both desminopathies and myotilinopathies, characterized by mutation of desmin and myotilin, respectively [52]. Abnormal modifications have often been observed in the aggregated proteins, supporting the aggregation mechanisms regulated by post-translational modifications. In a model of Huntington's disease, oxidation of methionine residues in the abnormal huntingtin protein occurs only in the aggregated protein, but not in the soluble state. Methionine oxidation creates additional interactions among huntingtin aggregates and alters their overall morphlogies [53].

These modifications of desmin can be considered as toxic effects, and may impair degradation pathways, including ubiquitin-proteasomal function. Mildly oxidized proteins are degraded by the proteasome, but evidence suggests that moderately or heavily oxidized proteins are rather degraded by autophagy. However, it has been reported that greatly modified substrates that are incompletely degraded accumulate within the lysosomal compartments, resulting in the formation of lipofuscin-like and autofluorescent aggregates [6]. Accumulation eventually results in impaired turnover of autophagy of proteins as well as of large organelles such as damaged proteasomal subunits or mitochondria.

Numerous studies using rat, mouse, or primates have tested the efficacy of antioxidants on neurodegenerative diseases, such as Parkinson’s Alzheimer’s or Huntington's disease. Indeed, high doses of α-tocopherols, beta-carotene, ascorbic acid, and N-acetyl-cysteine, among a large variety of compounds with antioxidant properties, provide protection in these neurodegenerative models [54]. However, it is important to note that many of these compounds have other properties, and it should not be assumed that radical scavenging is their primary mode of action [55].

In addition, in MFMs, and in particular in desminopathies, typical signs of concomitant mitochondrial pathology are areas with accumulation or depletion of mitochondria with normal or abnormal morphologies [20, 21]. This situation could contribute to an increase in reactive oxygen species production, which could target aggregated desmin.

We demonstrate in this work that stimulation of macroautophagy reduces the proportion of cells with desmin aggregation by a mean value of ~50%, which is in accordance with results found in other models involving genes responsible for MFMs or coding for other intermediate filaments. In transgenic mice specifically expressing R120G mutant αB-crystallin in cardiac myocytes, a more than 2-fold increase in autophagic activity is triggered [56]. Mutations in GFAP (glial fibrillary acidic protein), the IF protein specific to astrocytes, lead to GFAP accumulation and induce macroautophagy [57]. Moreover, mutations in lamin A/C, a nuclear IF, induce abnormal desmin accumulation in myocytes, while treatment with rapamycin, which inhibits mTorC1 signaling and stimulates autophagy, decreases this accumulation [58].

Many of these disorders involve three complementary systems, constituting the main components of the PQC that prevent protein aggregation in normal cells: HSPs acting as chaperones to refold misfolded proteins; the UPS targeting and degrading mutant, damaged, or misfolded proteins; and the autophagic pathway [2]. A life-long steady decrease in PQC activity is proposed to be responsible for accumulation of abnormally folded proteins, formation of inclusion bodies, and development of neurodegenerative diseases such as Alzheimer’s, Parkinson’s, and Huntington's diseases (reviewed in [5]). This hypothesis may also explain why MFMs take decades to produce clinical signs, although with different kinetic parameters. The striking characteristic of these disorders is that, although cells contain significant levels of aggregation-prone proteins, disease does not become established immediately but is observed as the mature individual ages. In summary, age-related decline in overall proteolytic activity has been observed in almost all organisms and progressive intracellular accumulation of damaged proteins with age has been extensively documented.

In addition, progressive impairment of the PQC mechanisms may not be the only explanation for disorders characterized by protein aggregation. Aggregate-prone proteins may also be more resistant to the PQC mechanisms of protection than other cellular proteins. For example, with regard to autophagy, the reasons behind the expansion of the autophagic compartments seem to differ from one disease to another. It includes upregulation of autophagosome formation to cargo recognition failure, impaired fusion between autophagic vesicles and lysosomes, or inefficient degradation of the abnormal protein and resistance to autophagic clearance. In Parkinson’s disease for example, the protein α-synuclein inhibits autophagy [59]. In addition, expression of aggregation-prone proteins in cultured cells showed that those expressing a desmin mutant S13F are resistant to autophagic clearance and fail to recruit key components of the autophagic-lysosomal system (i.e., LAMP1, LC3, and mTor) [60]. In other neurodegenerative disorders, such as Alzheimer's disease, the massive accumulation of autophagic vacuoles is not associated with increased degradation, but the autophagic vacuoles contain visible cytosolic cargo that cannot be properly degraded. Finally, MFM caused by filamin C (FLNC) mutations exhibits altered expression of chaperone proteins and components of the proteasomal and autophagic degradation pathways in abnormal muscle fibers that harbor protein deposits, but not in neighboring muscle fibers without pathological protein aggregation. These findings suggest a dysfunction of protein stabilizing and degrading mechanisms that leads to a pathological accumulation of protein aggregates in abnormal fibers [61].

Because this human muscle disease takes decades to manifest clinical signs, it is difficult to attribute MFM pathology solely to a simple mechanistic explanation such as the alteration of desmin IF formation by one of the many desmin mutants. Therefore, a complex and multilevel concept of disease development, in which mutant desmin interferes with desmin interaction partners, signaling cascades, protein quality control systems, and the function of organelles, should be proposed. To confirm our findings, an animal model will be needed to test the therapeutic efficacy of treatments described in the cellular model.

## Materials and Methods

### Plasmids

For transient expression, previously generated human *desmin* wild-type or mutated cDNAs (Q389P and D399Y) [19, 26] were subcloned in *Eco*RI-*Bam*HI restriction sites of pLink expression vector (containing a 5’ c-myc tag). To generate a fusion construct between the green fluorescent protein and desmin, a NcoI-Klenow-XbaI fragment of the wild-type and mutant desmin coding sequence were cut from the pLink-Desmin construct and subcloned into an EGFP vector (Takara-Clontech) opened by BamHI-Klenow-XbaI. pcDNA3 was purchased from Life Technologies. Constructs expressing wild-type (WT) and dominant-negative (K298A) PAK1 were obtained from Pr. M. Kobb, University of Texas, USA; mDia dominant-negative (ΔN3) construct from Pr. S. Narumiya, Kyoto University, Japan; Rac1 WT and N17 dominant-negative constructs from Dr. K. J. Irani, the John Hopkins Hospital, USA; TAK1 WT from Dr. K. Matsumoto, Nagoya University, Japan. A PKCα WT-expressing construct was given by Dr C. V. Paya, Rochester Mayo Clinic, USA; Rho kinase from Pr. K. Kaibuchi, Nagoya University, Japan; PRAK dominant-negative from Dr. P. Sun, the Scripps Research Institute, USA. The double fluorescent vector pDest-mCherry-EGFP-LC3 was obtained from Pr. T. Johansen, University of Tromsø, Norway.

### Reagents

The different reagents were purchased from: 17DMAG (Invivogen), alpha-lipoic acid, alpha-tocopherol and *o*-acetyl-alpha-tocopherol, Trolox, Earl's balanced salts solution (EBSS), colchicine, curcumin, and dimethylsulphoxide (DMSO) (Sigma–Aldrich); Bafilomycin A1, KN93, PP242, Rapamycin (Calbiochem-Millipore), NSC23766 (Tocris).

### Cell lines, culture, and transfection

C2C12 cells (ATCC, CRL-1772) were grown in Dulbecco’s modified Eagle’s medium (DMEM, Life Technologies) supplemented with 10% fetal bovine serum (PAA) and 1% penicillin/streptomycin (Life Technologies). Stable clones were grown in DMEM, 20% fetal bovine serum, 1% penicillin/streptomycin, 1 mg/mL G418, and 2 μg/mL puromycin. Co-transfection of GFP-desmin and myc-Desmin constructs with various other genes in C2C12 cells was performed using the JetPEI method (Ozyme) according to the manufacturer’s instructions.

### Heat stress

The stable cell line expressing the myc-desmin D399Y mutant cDNA under control of a tetracycline-inducible promoter, named DesD399Y clone C26, was seeded at 3 x 10^3^ cells/cm^2^ and induced with Doxycycline (10 μg/mL) 24 h later. Heat stress was introduced 48 h after induction at 42°C for 2 h. Subsequently, the media was changed and cells analyzed 24 h later. Anti-aggregative compounds were tested by pre-treating cells for 16 h before stress with PP242 (10 μM).

### Immunohistochemistry

Primary antibodies: mouse monoclonal anti-c-Myc antibody (Santa Cruz Biotechnologies; 1/100) and isotype specific secondary antibodies with anti-mouse and anti-rabbit Alexa-568 or -488 (Molecular Probes) were used. For GFP immunofluorescence, cells were fixed on slides with 3% paraformaldehyde for 10 min at room temperature, washed in PBS, and mounted in Fluoromount medium (Interchim). For anti-c-Myc immunofluorescence, cells were fixed with 70% methanol/30% acetone for 7 min at 4°C, washed with PBS, saturated with 10% fetal bovine serum for 30 min at room temperature, and incubated with the anti-c-Myc primary mouse monoclonal antibody (Santa Cruz) for 45 min at room temperature. Binding of primary antibodies was detected by incubating cells 45 min with suitable secondary antibodies. DNA was stained with Hoechst (1 μg/mL, Sigma-Aldrich) for 10 min. Finally, cells were washed in PBS and staining was visualized with confocal microscopy (ZEISS LSM700).

### Western blotting

Proteins were extracted using Tris-HCl buffer 0.1 M pH 7.5 that contained 1 mM EDTA, 150 mM NaCl, 0.1% NP40, 0.1 mM Na orthovanadate, 2 mM DTT, and 1 mM PMSF (lysis buffer), separated by SDS-PAGE, and transferred to nitrocellulose membranes (Macherey Nagel), which were then incubated with 5% non-fat milk in PBS-1% Tween. Primary antibody was added at the appropriate dilution and membranes were incubated for 16 h at 4°C. Primary antibodies used were: (1) rabbit polyclonal antibody anti-PKC-alpha (Cell Signaling Technology, 1/1000); (2) rabbit polyclonal antibody anti-Rac(1/2/3) (Cell Signaling Technology, 1/1000); (3) mouse monoclonal anti-c-Myc antibody (Santa Cruz Biotechnologies, 1/1000); (4) rabbit polyclonal antibody anti-HA-probe (Santa Cruz, 1/1000); (5) mouse monoclonal anti-alpha-actin (Millipore, 1/2000); (6) rabbit polyclonal antibody anti-LC3 (Sigma-Aldrich, 1/1000); (7) rabbit polyclonal antibody anti-GFP (Invitrogen, 1/1000); isotype-specific secondary antibody coupled with a horseradish peroxidase (Pierce, 1/2000) was detected by incubating with ECL+ (GE Healthcare) and visualized with CCD camera (FUJI Las 4000).

### Flow cytometry assays

Cells were seeded on 6-well plates at 3 x 10^3^ cells/cm^2^ and transfected the next day for 4 hours with GFP-desmin-expressing constructs to generate aggregates, washed, grown for 16 h, and simultaneously treated with various reagents. Cells were then trypsinized and resuspended in Annexin V binding buffer (Biolegend). GFP-Desmin, propidium iodide (BD Biosciences), and APC-Annexin V (Biolegend) were detected using the 488-nm (GFP and Propidium Iodide) and 633-nm (APC) excitation laser and a 530/30-nm, 610/20 and 660/20-nm bandpass emission filter respectively in FACS Aria II (BD Biosciences). A total of 15,000 events were collected for each analysis

### Counting and statistical analysis

Images are tile scan 5x5 on 3 random nuclear fields chosen on Hoechst-stained areas with a 10x objective. Total cell number was calculated by counting nuclei with an ImageJ (NIH, Washington, USA) software in-house macro. Cells presenting aggregates were visually counted on the images. Experiment was repeated at least 3 times. Statistical analysis of the results was performed with Kruskal and Wallis nonparametric tests and R software. Significant differences were accepted at p < 0.05.

## Supporting Information

S1 Fig Kinetics of myc-tagged desmin WT and D399Y mutant aggregation. C2C12 murine myoblasts transiently transfected with expression vectors coding for a myc-tagged desmin WT or the mutant myc-Desmin D399Y were fixed at various times (4 to 80 h) following transfection, and myc-positive cells revealed. Surface areas of aggregates were measured on a panel of n = 30 cells in 3 independent experiments, and the mean value plotted against time. Error bars, s.e.m.
(TIF)

S2 Fig Efficient expression of PKC WT, Rac1 DN, and PAK1 WT in C2C12 cells. Cells were transiently transfected with PKC wildtype (WT; in Fig as PKC), Rac1 dominant-negative (DN; in Fig as Rac1), PAK1 WT (PAK1), PRAK DN (PRAK), TAK1 WT (TAK1), or pcDNA3 empty vector (CNTL). Sixteen h later, cells were lysed and cellular extracts analyzed in Western blots. Specific anti-PKC and anti-Rac antibodies were used, while for other constructs that were myc- or HA-tagged, anti-myc or anti-HA antibodies were used. In all cases, the control (CNTL) did not show a band for the kinase or the GTPase tested. All bands matched the expected size (arrowheads: PKC, 74 kDa; Rac1, 21 kDa; PAK1, 60 kDa; PRAK, 52 kDa; TAK1, 70 kDa).
(TIF)

S3 Fig Lack of toxicity associated with transfection of constructs modulating cell signaling pathways. C2C12 myoblasts were co-transfected with a pEGFP vector expressing the green fluorescent protein (GFP) together with the constructs indicated in Fig 2 (i.e., Rac1 WT, Rac1 DN, PAK1 WT, PAK1 DN, ROCK WT, mDia DN, PKC WT, PRAK DN and TAK1 WT). At 48 h following transfection, cells were fixed and GFP-positive cells were counted under microscope. Experiments were done 4 times independently (n = 2000 cells per condition for each experiment). No difference with the control (CNTL) pcDNA3 vector was found (p < 0.05 calculated with a non-parametric test).
(TIF)

S4 Fig Modulation of cell signaling pathways related to the cytoskeleton reduces desmin aggregation. (A) C2C12 cells were co-transfected with a GFP-tagged desmin WT and constructs coding for either wild type (WT) or dominant-negative mutant (DN) kinases or kinase-modulating proteins [i.e., Rac1, p21-activated protein kinase (PAK1), Rho kinase (ROCK), mammalian Diaphanous (mDia), protein kinase C (PKC), p38-regulated/activated protein kinase (PRAK) and transforming growth factor β activated kinase 1 (TAK1)]. At 20 h after transfection, cells were fixed and the total number of cells (n = 1000) and the number of transfected cells with aggregates were counted. Experiments were performed 4 times. The percentage of cells with aggregates is displayed on a box plot graph (Tukey's diagram). Asterisk indicates a result statistically different from the control co-transfected with the desmin mutant and the empty vector pcDNA3 (p < 0.05 calculated with a non-parametric test). (B) Same treatment as for (A) except that cells were transfected with myc-tagged constructs, desmin WT (left panel) and D399Y mutant (right panel). At 20 h after transfection, cells were fixed, revealed for myc-tagged desmin expression, and the number of transfected cells with or without aggregates were counted (n = 500). Experiments were performed 3 times.
(TIF)

S5 Fig No specific cell death for cells expressing GFP-desmin mutant and receiving α-tocopherol treatment. C2C12 cells were transfected with GFP-Desmin D399Y for 4 h, washed, and treated for 16 h with α-tocopherol (α-Toco, 300 μM), *O*-acetyl-α-tocopherol (Ac-α-Toco, 100 μM), or DMSO. Cells were then analyzed in a cell fluorimeter after probing with APC-annexin V and propidium iodide to estimate early apoptosis (EA), late apoptosis (LA), and necrosis (N). Values for apoptosis and necrosis were recorded for the whole cell population (TOTAL) as well for the GFP-positive cells (GFP+) representing transfected cells, which were around 20% of the total population. The ratio between the total cell death (EA + LA + N) for GFP+ cells, divided by the total cell death (EA + LA + N) for the total population (TOTAL) is indicated above bars for each treatment.
(TIF)

S6 Fig Screening for pharmaceutical activators of autophagy in myoblasts. (A) PP242 is the best inducer of autophagy in C2C12 myoblasts. Untransfected cells were treated for 5 h with the indicated compounds: temsirolimus (Tems, 20 nM), PP242 (10 μM), NF449 (200 μM), calpeptin (Calp, 50 μM), verapamil (Vera, 1 μM), and the solvent DMSO (CNTL). Cell extracts were analyzed in Western blot experiments for expression of endogenous LC3, and for actin as a loading control. The quantity of endogenous LC3-II reflects the level of autophagy, when the process is not blocked. Levels of activation of autophagy were obtained by quantification of the LC3-II (anti-LC3 antibody) band normalized to actin (anti-actin antibody), as recommended in [62], with the control value (right CNTL) assigned as 1.0. This figure is representative of 3 independent experiments. (B) Comparison of PP242 with various inducers of autophagy. All treatments were applied for 5 h, after which cells were lysed and analyzed via Western blotting for LC3 activation, as described above. Rapamycin (270 nM), or amino acid and glucose nutrient deprivation in EBSS medium serve as inducers of autophagy. Bafilomycin (200 nM) inhibits autophagososme acidification, and thus fusion with the lysosome, which results in the accumulation of vesicles. It gives the maximal value of LC3-II because it is no longer degraded nor recycled during 5 h of treatment. Compared to rapamycin (1.5-fold activation) or nutrient deprivation, treatment with PP242 for 5 h gives the highest value of induction (2.5-fold, right panel). This figure is representative of 3 independent experiments. (C) Control of autophagosome turnover. C2C12 cells were transiently transfected with a construct expressing LC3 fused to mCherry (red) and GFP (green) for 4 h (CG-LC3), washed, and treated with rapamycin (270 nM), bafilomycin (200 nM), PP242 (10 μM), or deprived of nutrients for 5 h in EBSS. Following fixation, cells were visualized under a microscope. Autophagosomes appear as yellow dots (red + green), while autophagolysosomes, once fused to lysosomes and acidified, appear as red dots. With bafilomycin, no acidification occurred and only yellow vesicles accumulated, while with PP242, more red dots indicate a higher turnover. Scale bar, 10 μm.
(TIF)

S7 Fig No specific toxicity of PP242 in muscular cells expressing desmin mutants. C2C12 cells were transfected with GFP-Desmin D399Y for 4 h, washed, and treated for 16 h with PP242 (10 μM). Cells were then analysed in a cell fluorimeter after probing with APC-annexin V and propidium iodide to estimate early apoptosis (EA), late apoptosis (LA) and necrosis (N). Values for apoptosis and necrosis were recorded for the whole cellular population (TOTAL) as well for the GFP-positive cells (GFP+). GFP+ cells represent transfected cells, which were around 20% of the total population. Ratios between values of cell death (EA + LA + N) for GFP+ cells, divided by values of cell death (AE + LA + N) for the total population (TOTAL) are indicated above bars, for each treatment. The figure represents results from 4 independent experiments.
(TIF)

S8 Fig Validation of the GFP-LC3 vector as a probe for monitoring autophagy. In the left panel, the GFP-LC3-expressing vector was co-transfected with the Rac1-expressing construct or the empty vector pcDNA3 for 4 h. To confirm results obtained in Fig 6 with an anti-GFP antibody, the Western blot obtained with cellular extracts was stained with an anti-LC3 antibody. A pattern similar to that for Fig 6 was obtained. In the middle panel, cells were transfected with the pEGFP vector expressing only GFP, with GFP-LC3, or with pcDNA3 as a negative control, to check for efficiency and specificity of the anti-GFP antibody. In the right panel, C2C12 cells were transiently transfected with a GFP-LC3-expressing construct for 4 h, washed, and treated for 5 h with rapamycin (Rapa, 270 nM), bafilomycin (Bafilo, 200 nM), PP242 (10 μM), or (DMSO). All three positive control treatments induced significantly higher LC3-II levels compared to CNTL.
(TIF)

S9 Fig Autophagy inducers and antioxidants cooperate to reduce aggregation in C2C12 myoblasts. (A) Expression of Rac1 DN protein and treatment with PP242 cooperate to reduce desmin aggregates. C2C12 cells were co-transfected with the myc-Desmin WT (left panel) and myc-Desmin D399Y (right panel) expressing vectors and Rac1 DN or pcDNA3 (CNTL and PP242) for 4 h. Cells were washed and subsequently incubated with PP242 (5 μM) or DMSO for 16 h. Cells were then fixed and the numbers of cells with aggregates were counted under a microscope (n = 200). A box plot representing 3 independent experiments is shown. Statistical analysis showed significant differences from pcDNA3 (p < 0.05 with a non-parametric test) as indicated by an asterisk. A significant difference for Rac1 DN + PP242 treatment versus either PP242 or Rac1 DN alone is indicated by an asterisk above an horizontal bar (p < 0.01). (B) PP242 and α-tocopherols cooperate to reduce desmin aggregation. C2C12 cells were transiently transfected with a myc-Desmin WT (left panel) or a myc-Desmin D399Y (right panel) vector for 4 h. They were washed and then treated with PP242 (5 μM), α-tocopherol (α-Toco, 150 μM), or both for 16 h. The box plot represents 3 independent experiments (n = 200). An asterisk indicates a significant difference from control at p < 0.05, and an asterisk above the horizontal bar indicates a significant difference between double and single treatments (p < 0.05).
(TIF)

S10 Fig No specific toxicity of PP242 + NSC23766 + Trolox in muscular cells expressing desmin mutants. C2C12 cells were transfected with GFP-Desmin D399Y for 4 h, washed, and treated for 16 h with PP242 (5 μM), NSC23766 (50 μM), and Trolox (200 μM). Cells were then analyzed in a cell fluorimeter after probing with APC-annexin V and propidium iodide to estimate early apoptosis (EA), late apoptosis (LA), and necrosis (N). Values for apoptosis and necrosis were recorded for the whole cell population (TOTAL) as well for the GFP-positive cells (GFP+). GFP+ cells represent transfected cells, which were around 20% of the total population. Ratios between values of cell death (EA + LA + N) for GFP+ cells, divided by values of cell death (AE + LA + N) for the total population (TOTAL) are indicated above bars for each treatment.
(TIF)

S11 Fig Treatments with therapeutic drugs cooperate to reduce desmin aggregation. (A) C2C12 cells were transiently transfected with the myc-Desmin WT or D399Y-expressing vectors for 4 h, washed, and subsequently incubated with PP242 (5 μM), NSC23766 (50 μM), Trolox (200 μM), all three compounds, or DMSO (CNTL) for 16 h. Cells were then fixed and counted under a microscope for the percentage having aggregates. A box plot representing 3 independent experiments is shown (n = 100). Statistical analysis with a non-parametric test revealed significant differences indicated by an asterisk (p < 0.05), and an asterisk above the horizontal bar indicates a significant difference between double and single treatments (p < 0.05). (B) C2C12 cells stably transfected and expressing the myc-Desmin D399Y mutant construct under a Doxycycline-inducible promoter (Tet-ON system), were treated with DMSO, PP242, NSC23766, Trolox or a combination of these products, in the presence of Doxycycline for 16 h. Cells were then subjected to heat-shock at 42°C for 2 h (HS), the medium was changed, and cells were incubated in normal growth medium for 24 h. Cells were then fixed and aggregates were counted under a microscope (n = 400 total cells in each 3 independent experiments–for "PP242 + Trolox", 1 experiment). Asterisk indicates a result statistically different from the control (p < 0.05 calculated with a non-parametric test).
(TIF)

S1 Table List of cDNA constructs tested for inhibition of desmin aggregation in a first round of screening. C2C12 cells were transfected with the construct GFP-Desmin D399Y and cDNA for 20 h before cell fixation. "-" means a reduction, " = ", no change, and "+" indicates an increase in desmin mutant aggregation. The experiments were performed 3 times.
(DOC)

S2 Table List of pharmacological compounds tested for inhibition of desmin aggregation in a first round of screening. Doses were choosen according to current values found in the literature. C2C12 cells were transfected with the construct GFP-Desmin D399Y for 4 h, and compounds were added for 16 h before cell fixation. "-" means a reduction, " = ", no change, and "+" indicates an increase in desmin mutant aggregation. The experiments were performed 2 times.
(DOC)

S3 Table List of pharmacological products used to stimulate autophagy in C2C12 cells. Various products described in the literature were tested for their capacity to enhance cellular autophagy in C2C12 cells. Following 5 h of treatment, cells were lysed and LC3 processing analyzed on Western blots. Values of fold-change in induction of LC3-II normalized to actin are the results of 3 independent experiments.
(DOC)

S4 Table Summary of the percentage of reduction of desmin aggregates obtained with various desmin constructs and treatments in C2C12 myoblasts. Values were calculated form the difference between the percentages of aggregates in the control and the treated points, divided by the control percentage, and expressed as a percentage. Values were taken from the different figures in the present work and from data not shown. When several values were available, the highest one was presented.
(DOC)
