# Supplementary figures and images for: Antioxidant Treatment and Induction of Autophagy Cooperate to Reduce Desmin Aggregation in a Cellular Model of Desminopathy
Source: PLoS One. 2015 Sep 2;10(9):e0137009. doi: 10.1371/journal.pone.0137009 (PMC4557996; doi:10.1371/journal.pone.0137009)

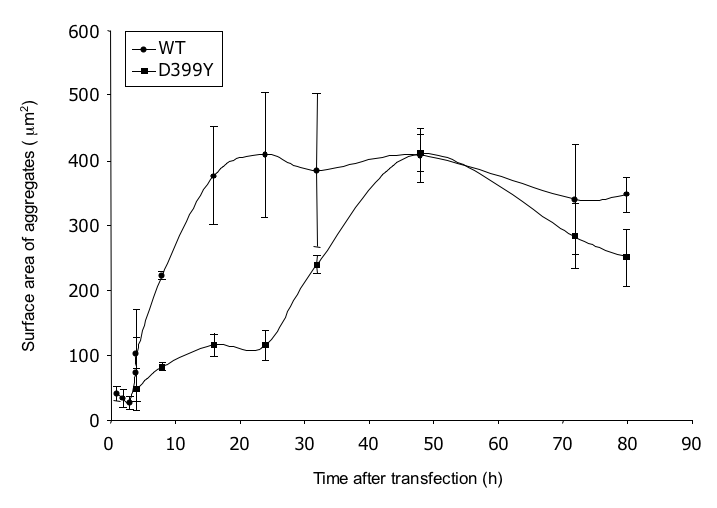

Supplement: S1 Fig — C2C12 murine myoblasts transiently transfected with expression vectors coding for a myc-tagged desmin WT or the mutant myc-Desmin D399Y were fixed at various times (4 to 80 h) following transfection, and myc-positive cells revealed. Surface areas of aggregates were measured on a panel of n = 30 cells in 3 independent experiments, and the mean value plotted against time. Error bars, s.e.m. (TIF) [file pone.0137009.s001.tif]

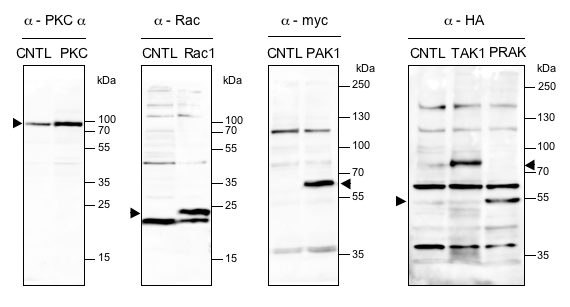

Supplement: S2 Fig — Cells were transiently transfected with PKC wildtype (WT; in Fig as PKC), Rac1 dominant-negative (DN; in Fig as Rac1), PAK1 WT (PAK1), PRAK DN (PRAK), TAK1 WT (TAK1), or pcDNA3 empty vector (CNTL). Sixteen h later, cells were lysed and cellular extracts analyzed in Western blots. Specific anti-PKC and anti-Rac antibodies were used, while for other constructs that were myc- or HA-tagged, anti-myc or anti-HA antibodies were used. In all cases, the control (CNTL) did not show a band for the kinase or the GTPase tested. All bands matched the expected size (arrowheads: PKC, 74 kDa; Rac1, 21 kDa; PAK1, 60 kDa; PRAK, 52 kDa; TAK1, 70 kDa). (TIF) [file pone.0137009.s002.tif]

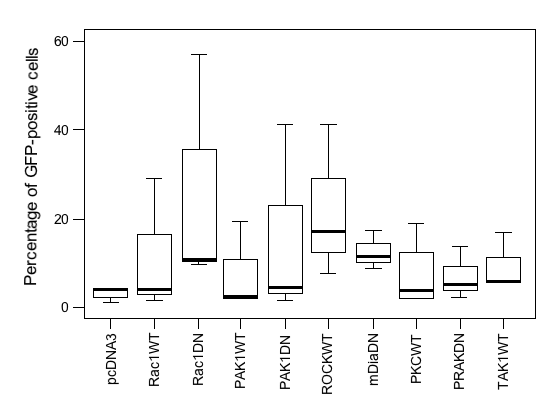

Supplement: S3 Fig — C2C12 myoblasts were co-transfected with a pEGFP vector expressing the green fluorescent protein (GFP) together with the constructs indicated in Fig 2 (i.e., Rac1 WT, Rac1 DN, PAK1 WT, PAK1 DN, ROCK WT, mDia DN, PKC WT, PRAK DN and TAK1 WT). At 48 h following transfection, cells were fixed and GFP-positive cells were counted under microscope. Experiments were done 4 times independently (n = 2000 cells per condition for each experiment). No difference with the control (CNTL) pcDNA3 vector was found (p < 0.05 calculated with a non-parametric test). (TIF) [file pone.0137009.s003.tif]

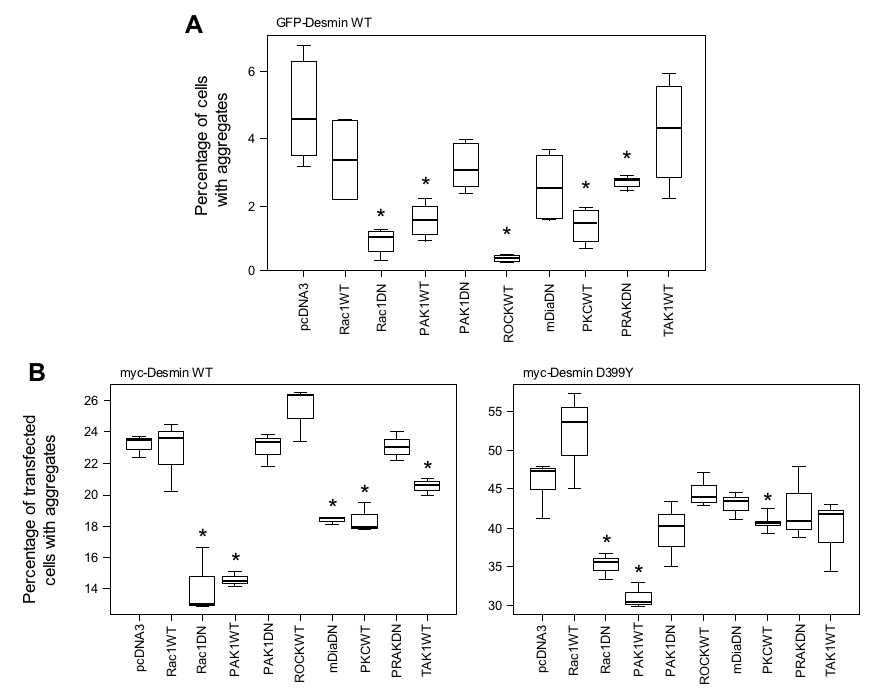

Supplement: S4 Fig — (A) C2C12 cells were co-transfected with a GFP-tagged desmin WT and constructs coding for either wild type (WT) or dominant-negative mutant (DN) kinases or kinase-modulating proteins [i.e., Rac1, p21-activated protein kinase (PAK1), Rho kinase (ROCK), mammalian Diaphanous (mDia), protein kinase C (PKC), p38-regulated/activated protein kinase (PRAK) and transforming growth factor β activated kinase 1 (TAK1)]. At 20 h after transfection, cells were fixed and the total number of cells (n = 1000) and the number of transfected cells with aggregates were counted. Experiments were performed 4 times. The percentage of cells with aggregates is displayed on a box plot graph (Tukey's diagram). Asterisk indicates a result statistically different from the control co-transfected with the desmin mutant and the empty vector pcDNA3 (p < 0.05 calculated with a non-parametric test). (B) Same treatment as for (A) except that cells were transfected with myc-tagged constructs, desmin WT (left panel) and D399Y mutant (right panel). At 20 h after transfection, cells were fixed, revealed for myc-tagged desmin expression, and the number of transfected cells with or without aggregates were counted (n = 500). Experiments were performed 3 times. (TIF) [file pone.0137009.s004.tif]

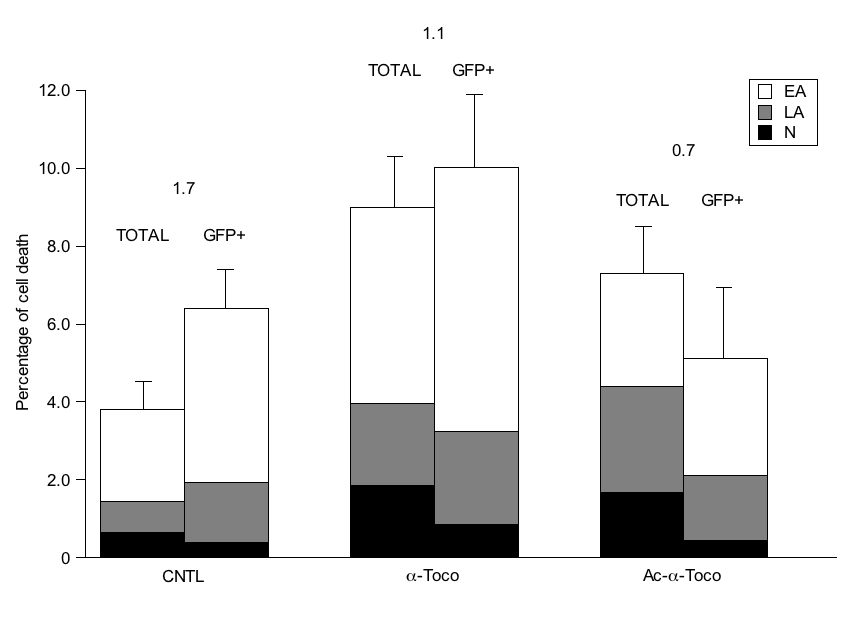

Supplement: S5 Fig — C2C12 cells were transfected with GFP-Desmin D399Y for 4 h, washed, and treated for 16 h with α-tocopherol (α-Toco, 300 μM), O-acetyl-α-tocopherol (Ac-α-Toco, 100 μM), or DMSO. Cells were then analyzed in a cell fluorimeter after probing with APC-annexin V and propidium iodide to estimate early apoptosis (EA), late apoptosis (LA), and necrosis (N). Values for apoptosis and necrosis were recorded for the whole cell population (TOTAL) as well for the GFP-positive cells (GFP+) representing transfected cells, which were around 20% of the total population. The ratio between the total cell death (EA + LA + N) for GFP+ cells, divided by the total cell death (EA + LA + N) for the total population (TOTAL) is indicated above bars for each treatment. (TIF) [file pone.0137009.s005.tif]

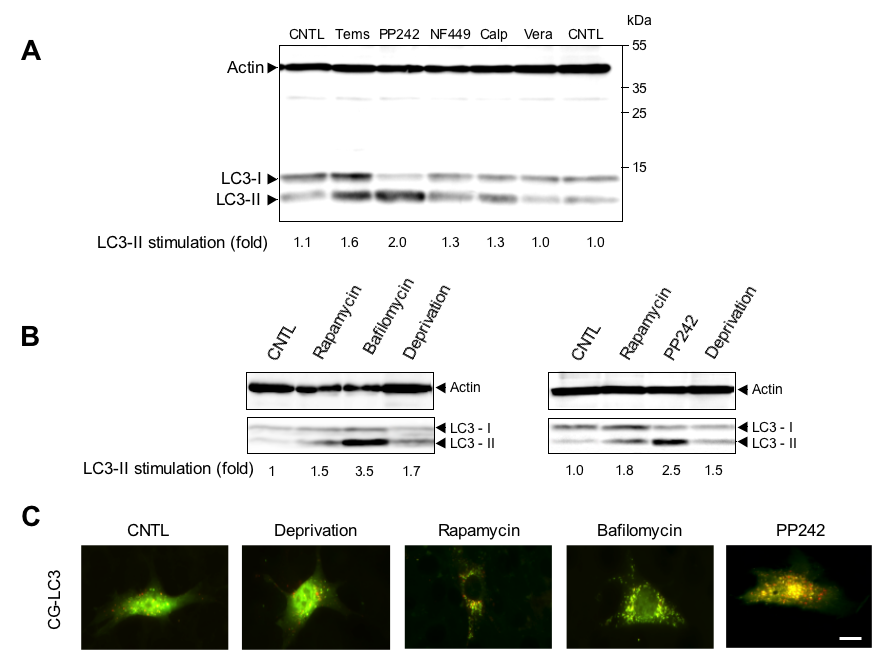

Supplement: S6 Fig — (A) PP242 is the best inducer of autophagy in C2C12 myoblasts. Untransfected cells were treated for 5 h with the indicated compounds: temsirolimus (Tems, 20 nM), PP242 (10 μM), NF449 (200 μM), calpeptin (Calp, 50 μM), verapamil (Vera, 1 μM), and the solvent DMSO (CNTL). Cell extracts were analyzed in Western blot experiments for expression of endogenous LC3, and for actin as a loading control. The quantity of endogenous LC3-II reflects the level of autophagy, when the process is not blocked. Levels of activation of autophagy were obtained by quantification of the LC3-II (anti-LC3 antibody) band normalized to actin (anti-actin antibody), as recommended in [62], with the control value (right CNTL) assigned as 1.0. This figure is representative of 3 independent experiments. (B) Comparison of PP242 with various inducers of autophagy. All treatments were applied for 5 h, after which cells were lysed and analyzed via Western blotting for LC3 activation, as described above. Rapamycin (270 nM), or amino acid and glucose nutrient deprivation in EBSS medium serve as inducers of autophagy. Bafilomycin (200 nM) inhibits autophagososme acidification, and thus fusion with the lysosome, which results in the accumulation of vesicles. It gives the maximal value of LC3-II because it is no longer degraded nor recycled during 5 h of treatment. Compared to rapamycin (1.5-fold activation) or nutrient deprivation, treatment with PP242 for 5 h gives the highest value of induction (2.5-fold, right panel). This figure is representative of 3 independent experiments. (C) Control of autophagosome turnover. C2C12 cells were transiently transfected with a construct expressing LC3 fused to mCherry (red) and GFP (green) for 4 h (CG-LC3), washed, and treated with rapamycin (270 nM), bafilomycin (200 nM), PP242 (10 μM), or deprived of nutrients for 5 h in EBSS. Following fixation, cells were visualized under a microscope. Autophagosomes appear as yellow dots (red + green), while autophagolysosomes, o [file pone.0137009.s006.tif]

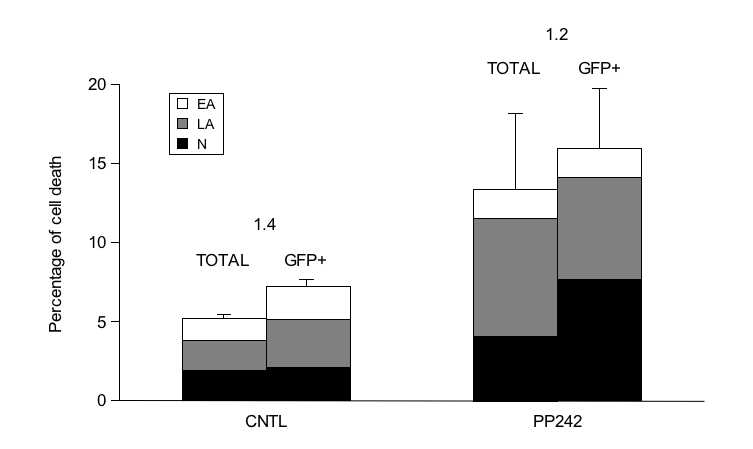

Supplement: S7 Fig — C2C12 cells were transfected with GFP-Desmin D399Y for 4 h, washed, and treated for 16 h with PP242 (10 μM). Cells were then analysed in a cell fluorimeter after probing with APC-annexin V and propidium iodide to estimate early apoptosis (EA), late apoptosis (LA) and necrosis (N). Values for apoptosis and necrosis were recorded for the whole cellular population (TOTAL) as well for the GFP-positive cells (GFP+). GFP+ cells represent transfected cells, which were around 20% of the total population. Ratios between values of cell death (EA + LA + N) for GFP+ cells, divided by values of cell death (AE + LA + N) for the total population (TOTAL) are indicated above bars, for each treatment. The figure represents results from 4 independent experiments. (TIF) [file pone.0137009.s007.tif]

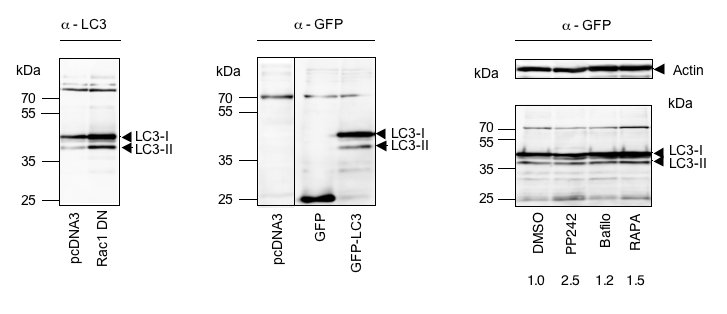

Supplement: S8 Fig — In the left panel, the GFP-LC3-expressing vector was co-transfected with the Rac1-expressing construct or the empty vector pcDNA3 for 4 h. To confirm results obtained in Fig 6 with an anti-GFP antibody, the Western blot obtained with cellular extracts was stained with an anti-LC3 antibody. A pattern similar to that for Fig 6 was obtained. In the middle panel, cells were transfected with the pEGFP vector expressing only GFP, with GFP-LC3, or with pcDNA3 as a negative control, to check for efficiency and specificity of the anti-GFP antibody. In the right panel, C2C12 cells were transiently transfected with a GFP-LC3-expressing construct for 4 h, washed, and treated for 5 h with rapamycin (Rapa, 270 nM), bafilomycin (Bafilo, 200 nM), PP242 (10 μM), or (DMSO). All three positive control treatments induced significantly higher LC3-II levels compared to CNTL. (TIF) [file pone.0137009.s008.tif]

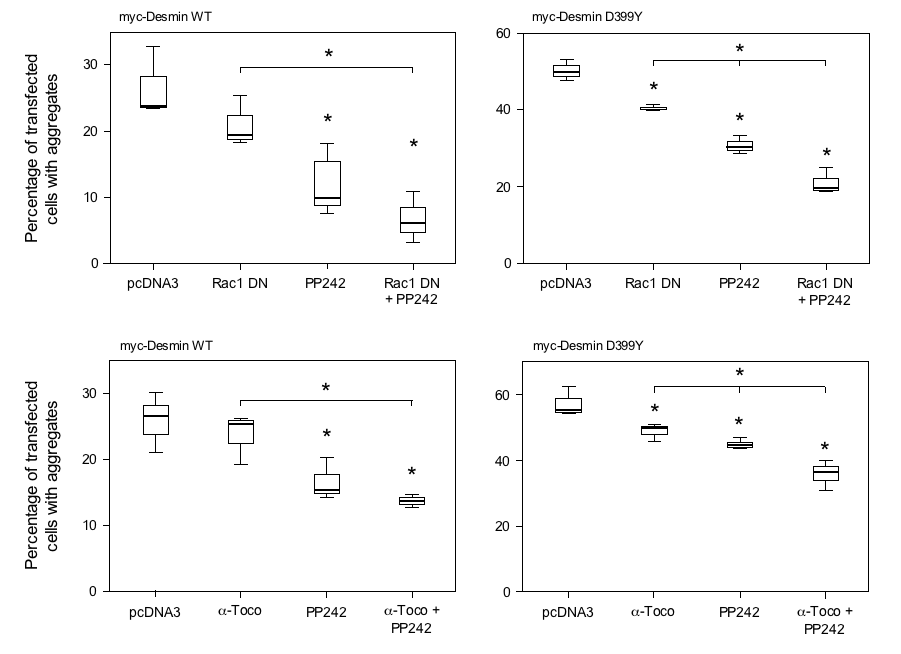

Supplement: S9 Fig — (A) Expression of Rac1 DN protein and treatment with PP242 cooperate to reduce desmin aggregates. C2C12 cells were co-transfected with the myc-Desmin WT (left panel) and myc-Desmin D399Y (right panel) expressing vectors and Rac1 DN or pcDNA3 (CNTL and PP242) for 4 h. Cells were washed and subsequently incubated with PP242 (5 μM) or DMSO for 16 h. Cells were then fixed and the numbers of cells with aggregates were counted under a microscope (n = 200). A box plot representing 3 independent experiments is shown. Statistical analysis showed significant differences from pcDNA3 (p < 0.05 with a non-parametric test) as indicated by an asterisk. A significant difference for Rac1 DN + PP242 treatment versus either PP242 or Rac1 DN alone is indicated by an asterisk above an horizontal bar (p < 0.01). (B) PP242 and α-tocopherols cooperate to reduce desmin aggregation. C2C12 cells were transiently transfected with a myc-Desmin WT (left panel) or a myc-Desmin D399Y (right panel) vector for 4 h. They were washed and then treated with PP242 (5 μM), α-tocopherol (α-Toco, 150 μM), or both for 16 h. The box plot represents 3 independent experiments (n = 200). An asterisk indicates a significant difference from control at p < 0.05, and an asterisk above the horizontal bar indicates a significant difference between double and single treatments (p < 0.05). (TIF) [file pone.0137009.s009.tif]

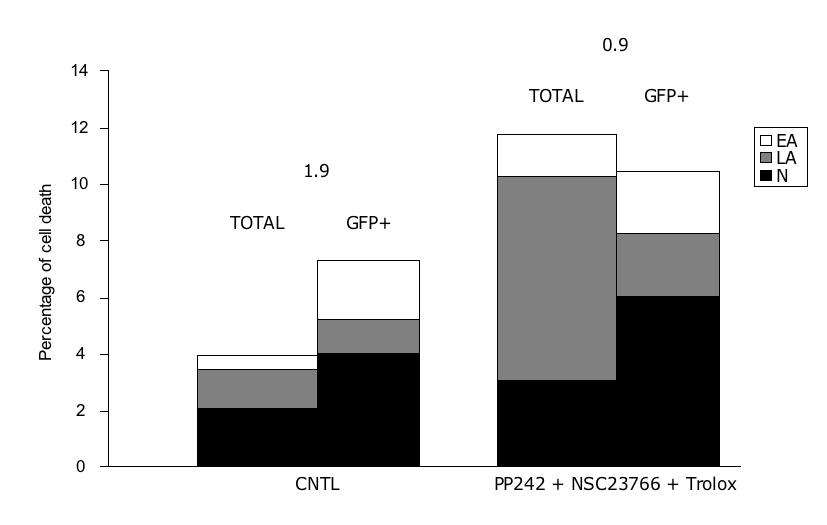

Supplement: S10 Fig — C2C12 cells were transfected with GFP-Desmin D399Y for 4 h, washed, and treated for 16 h with PP242 (5 μM), NSC23766 (50 μM), and Trolox (200 μM). Cells were then analyzed in a cell fluorimeter after probing with APC-annexin V and propidium iodide to estimate early apoptosis (EA), late apoptosis (LA), and necrosis (N). Values for apoptosis and necrosis were recorded for the whole cell population (TOTAL) as well for the GFP-positive cells (GFP+). GFP+ cells represent transfected cells, which were around 20% of the total population. Ratios between values of cell death (EA + LA + N) for GFP+ cells, divided by values of cell death (AE + LA + N) for the total population (TOTAL) are indicated above bars for each treatment. (TIF) [file pone.0137009.s010.tif]

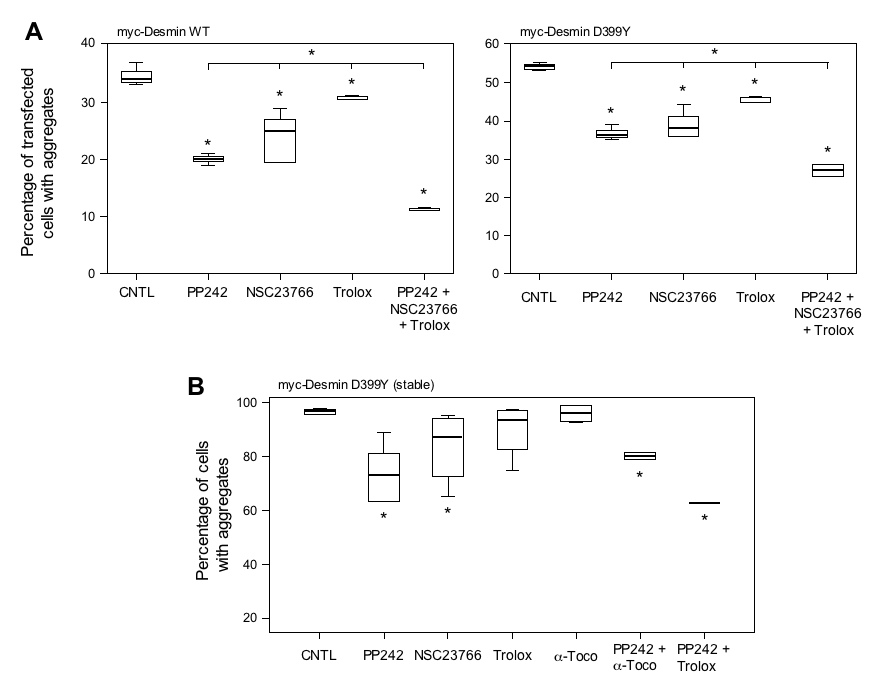

Supplement: S11 Fig — (A) C2C12 cells were transiently transfected with the myc-Desmin WT or D399Y-expressing vectors for 4 h, washed, and subsequently incubated with PP242 (5 μM), NSC23766 (50 μM), Trolox (200 μM), all three compounds, or DMSO (CNTL) for 16 h. Cells were then fixed and counted under a microscope for the percentage having aggregates. A box plot representing 3 independent experiments is shown (n = 100). Statistical analysis with a non-parametric test revealed significant differences indicated by an asterisk (p < 0.05), and an asterisk above the horizontal bar indicates a significant difference between double and single treatments (p < 0.05). (B) C2C12 cells stably transfected and expressing the myc-Desmin D399Y mutant construct under a Doxycycline-inducible promoter (Tet-ON system), were treated with DMSO, PP242, NSC23766, Trolox or a combination of these products, in the presence of Doxycycline for 16 h. Cells were then subjected to heat-shock at 42°C for 2 h (HS), the medium was changed, and cells were incubated in normal growth medium for 24 h. Cells were then fixed and aggregates were counted under a microscope (n = 400 total cells in each 3 independent experiments–for "PP242 + Trolox", 1 experiment). Asterisk indicates a result statistically different from the control (p < 0.05 calculated with a non-parametric test). (TIF) [file pone.0137009.s011.tif]
