# Supplementary material for: Antioxidant Treatment and Induction of Autophagy Cooperate to Reduce Desmin Aggregation in a Cellular Model of Desminopathy
Source: PLoS One. 2015 Sep 2;10(9):e0137009. doi: 10.1371/journal.pone.0137009 (PMC4557996; doi:10.1371/journal.pone.0137009)
Supplement: S1 Table — C2C12 cells were transfected with the construct GFP-Desmin D399Y and cDNA for 20 h before cell fixation. "-" means a reduction, " = ", no change, and "+" indicates an increase in desmin mutant aggregation. The experiments were performed 3 times. (DOC) [file pone.0137009.s012.doc]

**S1 Table**. List of cDNA constructs tested for inhibition of desmin aggregation in a first round of screening.

|  | Name | Function | Aggregates |
| --- | --- | --- | --- |
| 1 | cdc42 CA (V12) | Small GTPase of the Rho, Rac, cdc42 familly | = |
| 2 | cdc42 DN (V17) | " | = |
| 3 | Rac 1 WT | Small GTPase of the Rho, Rac, cdc42 familly | -/= |
| 4 | Rac1 DN | " | - |
| 5 | Rho DN | Small GTPase of the Rho, Rac, cdc42 familly | = |
| 6 | ROCK WT | Downstream effectors of Rho | - |
| 7 | Ras DN | Small GTPase | = |
| 8 | PAK 1 WT | p21-activated kinase 1 (PAK1), serine/threonine protein kinase | - |
| 9 | PAK1 DN | " | -/= |
| 10 | PKC WT | Protein kinase C (PKC) family of serine/threonine protein kinases | - |
| 11 | PKC DN | " | =/+ |
| 12 | mDia WT | Downstream effectors of Rho to actin | = |
| 13 | mDia DN | " | - |
| 14 | TAK 1 WT | Transforming growth factor beta activated kinase | - |
| 15 | TAK1 DN | " | = |
| 16 | GEFH 1 WT | RhoA guanine nucleotide exchange factors H1 | = |
| 17 | GEFH1 DN | " | = |
| 18 | PRAK WT | MAPK-activated protein kinase MK5 | = |
| 19 | PRAK DN | " | - |
| 20 | p190 WT | p190 Rho GTPase-activating protein | + |
| 21 | p190 DN | " | = |
| 22 | Ask1 DN | Apoptosis signal-regulated kinase 1 (MAP3K5) | = |
| 23 | Akt DN | Serine/threonine kinase activated downstream of integrin | + |
| 24 | Akt CA | " | = |
| 25 | p85 WT | p85 subunit of PI3-kinase | = |
| 26 | p85 DN | " | + |
| 27 | p38 WT | Mitogen-activated protein kinase (MAPK) | + |
| 28 | p38 DN | " | = |
| 29 | MKK7 WT | MAPK kinase upstream of JNK MAPK | + |
| 30 | NF-κB WT |  | + |
| 31 | CaMKII CA | Calmodulin kinase | + |

C2C12 cells were transfected with the construct GFP-Desmin D399Y and cDNA for 20 h before cell fixation. "-" means a reduction, "=", no change, and "+" indicates an increase in desmin mutant aggregation. The experiments were performed 2 times.
