# Supplementary material for: Antioxidant Treatment and Induction of Autophagy Cooperate to Reduce Desmin Aggregation in a Cellular Model of Desminopathy
Source: PLoS One. 2015 Sep 2;10(9):e0137009. doi: 10.1371/journal.pone.0137009 (PMC4557996; doi:10.1371/journal.pone.0137009)
Supplement: S2 Table — Doses were choosen according to current values found in the literature. C2C12 cells were transfected with the construct GFP-Desmin D399Y for 4 h, and compounds were added for 16 h before cell fixation. "-" means a reduction, " = ", no change, and "+" indicates an increase in desmin mutant aggregation. The experiments were performed 2 times. (DOC) [file pone.0137009.s013.doc]

**S2 Table**. List of pharmacological compounds tested for inhibition of desmin aggregation in a first round of screening.

|  | Name | Aggregation | Function | Dose |
| --- | --- | --- | --- | --- |
| 1 | 17DMAG | - | HSP90 inhibitor/releases HSF1 | 20 nM |
| 2 | A23187 | = | Calcium ionophore | 1 μM |
| 3 | Ac-α-tocopherol | - | Antioxidant (Vitamin E) | 100 μM |
| 4 | α-lipoïc acid | - | Antioxidant | 300 μM |
| 5 | ALLM | = | Calpain and cathepsin inhibitor | 100 μM |
| 6 | ALLN | = | Calpain and cathepsin inhibitor | 100 μM |
| 7 | Anisomycin | - | JNK and p38 activator | 20 μM |
| 8 | α-tocopherol | - | Antioxidant | 300 μM |
| 9 | BAPTA AM | + | Intracellular calcium chelating agent | 50 μM |
| 10 | Bis-Indoyl-Maleimide | + | PKC inhibitor | 1 μM |
| 11 | Butanedione monoximine | - | Myosin ATP-ase inhibitor | 20 mM |
| 12 | Caffeic acid phenyl ester | = | Inhibits NF-kappaB transactivation | 30 μg/mL |
| 13 | Caffeine | = | Unloads calcium from ER | 1 mM |
| 14 | Calmidazolium | - | Calmodulin inhibitor | 5 μM |
| 15 | Calphostin C | - | PKC inhibitor | 1 mM |
| 16 | Cantharidin | = | Protein phosphatese 2A inhibitor | 1 μM |
| 17 | Colchicine | - | Microtubule polymerization inhibitor | 1 μM |
| 18 | Curcumin | - | Antiaggregative and antioxidant product | 5 μM |
| 19 | Cysclosporin A | + | Calcineurin inhibitor | 2 μg/mL |
| 20 | Diphenyleinoeidonium | = | NADPH oxidase inhibitor | 30 μM |
| 21 | Forkskolin | = | Activates cAMP formation | 100 mM |
| 22 | Gadolinium | + | Stretch-activated calcium channels inhibitor | 100 μM |
| 23 | Genistein | - | Tyrosine kinase inhibitor | 100 μM |
| 24 | Gö 6976 | = | Calcium-dependent PKC inhibitor | 3 μM |
| 25 | H89 | = | PKA inhibitor | 5 μM |
| 26 | Heparin | + | InP3 calcium receptors in the ER inhibitor | 50 μg/mL |
| 27 | Jasplakinolid | = | Stabilizer of F-actin | 10 μM |
| 28 | K252b | - | Tropomyosin receptor kinase/PKA inhibitor | 250 nM |
| 29 | KCl | = | Depolarizing transmembrane potential | 100 mM |
| 30 | KN62 | + | Calmodulin kinase II inhibitor | 200 μM |
| 31 | KN93 | - | Calmodulin kinase II inhibitor | 10 μM |
| 32 | Latrunculin | = | Actin polymerization inhibitor | 0.5 μM |
| 33 | L-NAME | = | Inhibitor of nitric oxyde production | 500 μM |
| 34 | LY294002 | = | PI3 kinase inhibitor | 25 μM |
| 35 | MG132 | + | Proteasome inhibitor | 5 μM |
| 36 | ML7 | + | Myosin light chain inhibitor | 300 μM |
| 37 | Nifedipine | = | Calcium channel L inhibitor | 10 μM |
| 38 | Nimodipine | + | Calcium channel L inhibitor | 10 μM |
| 39 | PBN | = | ROS inhibitor | 50 μM |
| 40 | PD098059 | = | Erk pathway inhibitor | 50 μM |
| 41 | PDTC | = | Antioxidant | 1 mM |
| 42 | Phenylarsine oxide | - | Tyrosine phosphatase inhibitor | 60 μg/mL |
| 43 | PP2 | = | Src family protein tyrosine kinase inhibitor | 50 μM |
| 44 | Rapamycin | - | mTor inhibitor/activator of autophagy | 200 nM |
| 45 | Ryanodine | = | Calcium receptors in the ER inhibitor | 2 μM |
| 46 | SB203580 | = | p38 MAPK inhibitor | 10 μM |
| 47 | SNAP | + | Nitric oxide donor | 100 μM |
| 48 | Taxol | = | Stabilizer of microtubules | 1 μM |
| 49 | TMB8 | + | Intracellular calcium chelating agent | 50 μM |
| 50 | Trehalose | = | mTor independent autophagy inducer | 10 mM |
| 51 | Vinblastine | + | Microtubule polymerization inhibitor | 1 μM |
| 52 | W7 | = | Calmodulin antagonist | 50 μM |
| 53 | ω-conotoxin | = | Specific calcium channel inhibitor | 5 μM |
| 54 | Wortmannin | - | PI3 kinase inhibitor | 2 μM |
| 55 | Y27632 | + | Rho kinase inhibitor | 5 μM |

Doses were choosen according to current values found in the literature. C2C12 cells were transfected with the construct GFP-Desmin D399Y for 4 h, and compounds were added for 16 h before cell fixation. "-" means a reduction, "=", no change, and "+" indicates an increase in desmin mutant aggregation. The experiments were performed 2 times.
