# Supplementary material for: Antioxidant Treatment and Induction of Autophagy Cooperate to Reduce Desmin Aggregation in a Cellular Model of Desminopathy
Source: PLoS One. 2015 Sep 2;10(9):e0137009. doi: 10.1371/journal.pone.0137009 (PMC4557996; doi:10.1371/journal.pone.0137009)
Supplement: S3 Table — Various products described in the literature were tested for their capacity to enhance cellular autophagy in C2C12 cells. Following 5 h of treatment, cells were lysed and LC3 processing analyzed on Western blots. Values of fold-change in induction of LC3-II normalized to actin are the results of 3 independent experiments. (DOC) [file pone.0137009.s014.doc]

**S3 Table**. List of pharmacological products used to stimulate autophagy in C2C12 cells.

| Name | Dose | LC3-II  induction | Function |
| --- | --- | --- | --- |
| Rapamycin | 250 nM | 1.9 | mTor inhibitor |
| Calpeptin | 50 μM | 2.12 | Calpain inhibitor |
| PP242 | 10 μM | 3.07 | mTor inhibitor |
| Deprivation | - | 1.5 | mTor inhibitor |
| Loperamide | 1 μM | 2.41 | L-type Calcium channels antogonist |
| Clonidine | 1 μM | 2.7 | Imidazoline receptor antagonist |
| Minoxidil | 1 μM | 1.25 | ATP-K+ channel activator |
| 2'5' dideoxyAdenosine | 200 μM | 1.14 | Adenylyl cyclase inhibitor |
| Temsirolimus | 20 nM | 1.6 | mTor inhibitor |
| NF449 | 200 μM | 2.02 | G-protein Gsa inhibitor |
| Verapamil | 1 μM | 1.03 | L-type Calcium channels antagonist |
| LiCl | 10 mM | 0.5 | GSK-3 inhibitor |

Various products described in the literature were tested for their capacity to enhance cellular autophagy in C2C12 cells. Following 5 h of treatment, cells were lysed and LC3 processing analyzed on Western blots. Values of fold-change in induction of LC3-II normalized to actin are the results of 3 independent experiments.
