# Supplementary material for: Antioxidant Treatment and Induction of Autophagy Cooperate to Reduce Desmin Aggregation in a Cellular Model of Desminopathy
Source: PLoS One. 2015 Sep 2;10(9):e0137009. doi: 10.1371/journal.pone.0137009 (PMC4557996; doi:10.1371/journal.pone.0137009)
Supplement: S4 Table — Values were calculated form the difference between the percentages of aggregates in the control and the treated points, divided by the control percentage, and expressed as a percentage. Values were taken from the different figures in the present work and from data not shown. When several values were available, the highest one was presented. (DOC) [file pone.0137009.s015.doc]

**S4 Table**. Summary of the percentage of reduction of desmin aggregates obtained with various desmin constructs and various treatments in C2C12 myoblasts.

| Treatment | Construct | Reduction of aggregation (%) |
| --- | --- | --- |
|  |  |  |
| Rac1 DN | GFP-Desmin WT | 76 |
|  | GFP-Desmin Q389P | 38 |
|  | GFP-Desmin D399Y | 35 |
|  | myc-Desmin WT | 39 |
|  | myc-Desmin D399Y | 23 |
| PAK1 WT | GFP-Desmin WT | 63 |
|  | GFP-Desmin Q389P | 18 |
|  | GFP-Desmin D399Y | 74 |
|  | myc-Desmin WT | 37 |
|  | myc-Desmin D399Y | 32 |
| PKC WT | GFP-Desmin WT | 65 |
|  | GFP-Desmin Q389P | 16 |
|  | GFP-Desmin D399Y | 30 |
|  | myc-Desmin WT | 20 |
|  | myc-Desmin D399Y | 15 |
| a-Tocopherol | GFP-Desmin WT | 65 |
|  | GFP-Desmin Q389P | 16 |
|  | GFP-Desmin D399Y | 65 |
|  | myc-Desmin WT | 16 |
|  | myc-Desmin D399Y | 15 |
|  | Stable myc-Desmin D399Y | 1 |
| PP242 | GFP-Desmin WT | 55 |
|  | GFP-Desmin Q389P | 44 |
|  | GFP-Desmin D399Y | 63 |
|  | myc-Desmin WT | 57 |
|  | myc-Desmin D399Y | 47 |
|  | Stable myc-Desmin D399Y | 33 |
| NSC23766 | GFP-Desmin WT | 11 |
|  | GFP-Desmin Q389P | ND |
|  | GFP-Desmin D399Y | 30 |
|  | myc-Desmin WT | 27 |
|  | myc-Desmin D399Y | 29 |
|  | Stable myc-Desmin D399Y | 10 |
| Trolox | GFP-Desmin WT | 16 |
|  | GFP-Desmin Q389P | ND |
|  | GFP-Desmin D399Y | 35 |
|  | myc-Desmin WT | 11 |
|  | myc-Desmin D399Y | 16 |
|  | Stable myc-Desmin D399Y | 4 |

The different values were calculated form the difference between the percentages of aggregates in the control and the treated points, divided by the control percentage, and expressend as a percentage. Values are taken from the different figures in the present work and from data not shown. When several values are available, the highest one is presented.
